# Understanding the dynamics of monomeric, dimeric, and tetrameric α‐synuclein structures in water

**DOI:** 10.1002/2211-5463.12069

**Published:** 2016-06-01

**Authors:** Jonathan Y. Mane, Maria Stepanova

**Affiliations:** ^1^ Department of Electrical and Computer EngineeringUniversity of AlbertaEdmontonCanada; ^2^ National Institute for NanotechnologyNational Research Council CanadaEdmontonCanada; ^3^ Department of Physics, Astronomy, and Materials ScienceMissouri State UniversitySpringfieldMOUSA

**Keywords:** dimers, folding dynamics, molecular dynamics simulations, oligomers, α‐synuclein, β‐sheet‐rich structures

## Abstract

Human α‐synuclein (αS) is an intrinsically disordered protein associated with Parkinson's disease. Molecular mechanisms of corruptive misfolding and aggregation of αS resulting in the disease, as well as the structure and other properties of the corresponding oligomers are not entirely understood yet, preventing the development of efficient therapies. In this study, we investigate the folding dynamics of initially unfolded hypothetical αS constructs in water using all‐atom molecular dynamics simulations. We also employ the novel essential collective dynamics method to analyze the results obtained from the simulations. Our comparative analysis of monomeric, dimeric, and tetrameric αS models reveals pronounced differences in their structure and stability, emphasizing the importance of small oligomers, particularly dimers, in the process of misfolding.

**Abbreviations**
ECD: essential collective dynamics
HH1: head‐to‐head #1 dimer
HH2: head‐to‐head #2 dimer
HH: head‐to‐head
HT1: head‐to‐tail #1 dimer
HT2: head‐to‐tail #2 dimer
HT: head‐to‐tail
MD: molecular dynamics
NAC: nonamyloid‐β component
NPT: isothermic‐isobaric molecular simulation ensemble in which the number of atoms, pressure, and temperature are held constant
NVT: canonical ensemble in which the number of atoms, volume, and temperature are held constant
PCA: principal components analysis
PDB: Protein Data Bank
PD: Parkinson's disease
rmsd: root‐mean‐square deviation
SPC: single point charge
αS: α‐synuclein

α‐Synuclein (αS) is a small cytoplasmic protein found mainly in the brain tissue and localized primarily in the nucleus and presynaptic terminals 1, 2. The biological function of α‐synuclein is not entirely clear yet, although it has been linked with neurotransmitter release and synaptic plasticity 3, 4, 5. αS is linked to Parkinson's disease (PD) and its etiology 6, 7, 8. In patients diagnosed with Parkinson's disease, abnormal intracellular deposits known as Lewy bodies and Lewy neurites are found 8, 9, 10. These deposits contain a large amount of αS suggesting that aggregation of αS might be involved in the pathogenesis of PD 10, although many details of the specific molecular mechanism are not clear yet.

The human αS is a 140‐amino acid protein. In micelle‐bound form, the secondary structure of αS consists of two noncontacting curved α‐helices connected by a short linker in an antiparallel arrangement as shown in Fig. 1. It can be seen as comprising of three regions—the N‐terminal, the central, and the C‐terminal regions, respectively. The N‐terminal region (residues 1–60) contains imperfect 11‐residue repeats with a highly conserved hexamer motif, KTK(E/Q)GV, which have been predicted to have a propensity to form amphipathic α‐helices 11, 12. The central region of αS, also known as the nonamyloid‐β component (NAC) domain (residues 61–95), is highly hydrophobic. The central part of the NAC domain, particularly a stretch of 12 amino acid residues, 71 through 82, is believed to be amyloidogenic and play an important role in the aggregation of αS 13, 14, 15, 16. It is followed by an extended and predominantly unstructured, highly mobile C‐terminal region (residues 96–140) 17, 18, 19. The C‐terminal region is predominantly acidic and contains high amounts of glutamate, aspartate, and proline residues. The C‐terminal region has a large net charge and low overall hydrophobicity, which is mainly responsible for the natively unfolded nature of αS 20. The 3D NMR structure of human micelle‐bound αS has been reported and deposited in the Protein Data Bank 21, PDB: 1XQ8 18.

**Figure 1:**
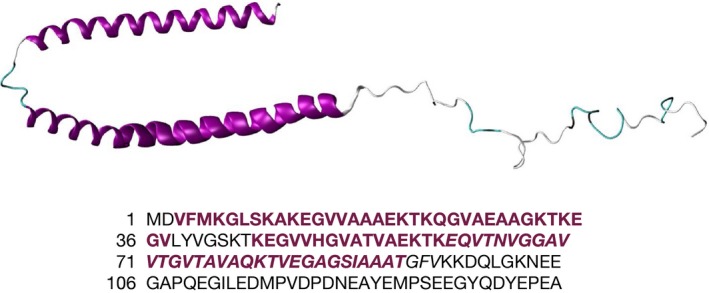
The structure and amino acid sequence of human micelle‐bound αS, according to PDB: 1XQ8 18. The two α‐helices are shown in purple (residues 3–37 and 45–92). The NAC region is found in the second α‐helix shown as a thicker purple and italicized in the sequence (residues 61–95). The remainder is the unstructured C‐terminal tail (residues 96–140).

The structural properties of αS are expected to play a key role in its biological function. In aqueous solutions, αS adopts a natively unfolded or intrinsically disordered conformation exhibiting a random‐coil secondary structure 22, 23. However, upon binding to lipid membranes, the generally unstructured αS adopts helical structures as shown in Fig. 1 23. A study has shown that purified αS exists mainly as an unfolded monomer 24. Other reports indicate that αS also may exist as a helically folded tetramer 25, 26, 27, 28. *In vitro* studies have demonstrated that monomeric αS can aggregate into several types of oligomeric species stabilized by β‐sheet interaction. These oligomeric species then form insoluble protofibrils, which in turn form amyloid fibrils resembling those found in Lewy bodies 29, 30, 31. The implication is that the transformation of αS from its native state into corruptive oligomers or protofibrils involves changes in its conformation accompanied by an increase in β‐sheet content 32. In particular, fluorescence resonance energy transfer (FRET) studies have provided interesting detail on the oligomerization of αS 33, 34, 35, 36. According to these studies, oligomerization of αS involves formation of intermediate partially folded multimeric species with properties distinct from those of both monomers and fibrils 33. The studies also indicate that the oligomerization is accompanied by a conformational change in αS 34, 35, which precedes the formation of β‐sheet‐rich species. 34. It was also demonstrated that several structural groups of αS oligomers may be formed as a result of the aggregation 35, 36. However, many aspects of the specific misfolding mechanisms, and their implication in αS oligomer formation, remain elusive.

Molecular dynamics (MD) modeling studies of the oligomerization of αS, its interaction with membranes, and aggregation of αS to form fibrils using molecular dynamics and docking tools have been reported 37, 38, 39. According to these studies, aggregation of monomeric αS to form oligomers may or may not occur, depending on the type of conformations αS dimers assume. Two types of conformations, a head‐to‐tail and a head‐to‐head dimeric conformation, were claimed to be involved in formation of the oligomers 37. The nonpropagating head‐to‐tail conformation was not found to favor further aggregation in the membrane. In contrast, the propagating head‐to‐head conformation, as the name implies, was shown to form multimers of αS. Studies involving the aggregation of αS to form fibrils normally focused on the NAC region, which is believed to play a crucial role in fibril formation 14, 15, 40. Although it is known that αS is intrinsically disordered in solution 22, 23, most of the published molecular dynamics studies address the micelle‐bound structure of αS with well‐defined α‐helical secondary structures. Complementing MD studies, Monte‐Carlo simulations 41 and Langevin dynamics modeling 42, 43 of intrinsically disordered αS chains have also been reported. Such models are potentially very efficient; however, they require a calibration 42, 43 or constraining 41 using experimentally determined structural data. Parameterization of coarse‐grained models toward ultimate predictive reliability remains in the pipeline.

In this work, we are interested in investigating how monomeric, dimeric, and tetrameric structures of αS may evolve in the course of an all‐atom MD simulation in aqueous solution by starting from a model built in the absence of any *a priori* secondary structures. Our aim is to monitor the evolution of new emerging secondary structures, particularly the formation β‐sheet structures, and compare the stability of these structures, in several hypothetical αS constructs starting from their respective unfolded configurations. We also report our analysis of the resulting structures obtained via MD simulations using the novel essential collective dynamics (ECD) methodology 44, 45, 46, 47, 48, 49, 50, 51, 52, 53.

## Materials and methods

### Materials

**Figure 2:**
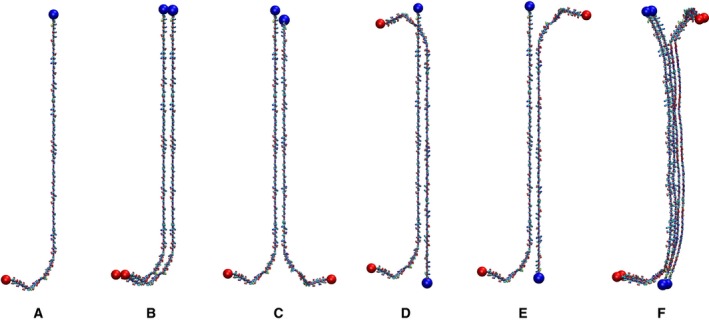
Starting configurations of the unfolded αS used in MD simulations: (A) monomer; (B) head‐to‐head #1 (HH1) dimer; (C) head‐to‐head #2 (HH2) dimer; (D) head‐to‐tail #1 (HT1); (E) head‐to‐tail #2 (HT2); (F) tetramer. N‐terminal and C‐terminal tails are indicated by blue and red spheres, respectively.

The sequence of human micelle‐bound αS protein molecule was obtained from the Protein Data Bank 21 (PDB: 1XQ8 18). In this work, the N‐terminal of αS has been denoted as the ‘head’ and the C‐terminal has been denoted as the ‘tail’. Using the primary protein sequence of 1XQ8, the model for the unfolded monomeric αS molecule was constructed with modeller v9.13 54, 55 (Fig. 2A). Next, the pymol 56 program was used to construct the unfolded dimer (Fig. 2B–E) and tetramer (Fig. 2F) models utilizing the unfolded monomer model previously created as the building block. The dimers were built by first duplicating the coordinates of the original monomer to produce a second monomer. Then a series of translations and/or rotations were performed on the second monomer coordinates aligning it to the first monomer to achieve the desired dimer configuration (Fig. 2B–E). For the tetramer, two units of the dimer (Fig. 2E) were stacked together such that the resulting model had the same interchain distances among the monomeric units. Molecular dynamics simulations were performed with gromacs software package version 4.6.5 57 using the OPLS‐AA 58 force field for the protein. All the secondary structure elements were identified by the dssp program, which employs backbone hydrogen bond patterns to discriminate among secondary structures 59. The timelines of secondary structures and distance maps were calculated employing the gromacs software package. Molecular graphics images were produced using the visual molecular dynamics (vmd) 60 program suite.

### Molecular dynamics simulations

We carried out molecular dynamics (MD) simulations to generate the trajectories for each of αS models. For each model system we performed three independent MD simulations. Each protein model was placed in a periodic triclinic box with a distance of 1.4 nm between the protein and the edges of the periodic box. Energy minimization in vacuum was performed on the protein using 1000 steps of the steepest descent algorithm in the presence of strong positional restraints. A force constant equal to 1 × 10^5^ kJ·mol^−1^·nm^−2^ was applied on all the heavy protein atoms to prevent large distortions to the protein structure by the vacuum environment. The minimized structure was then solvated with SPC 61 water molecules. Before the addition of counterions, the solvent around the protein was minimized using 1000 steps of the steepest descent algorithm in the presence of strong positional restraints. The same restraint values were used during the *in vacuo* minimization. This was done to prevent large distortions to the protein structure by the nonequilibrium solvent. Counterions (Na^+^ or Cl^−^) were then added to adjust the net charge of the system to zero. Six cycles of steepest descent minimization using 1000 steps each were then performed on the system with decreasing positional restraints on nonhydrogen protein atoms (*K*_posre_ = 10^5^, 10^4^, 10^3^, 10^2^, 10, and 0 kJ·mol^−1^·nm^−2^). Seven sequential steps of MD equilibration followed at a temperature of 310 K. The first six MD equilibration steps were performed with constant volume and temperature (NVT ensemble) while decreasing the positional restraints on the nonhydrogen protein atoms, the same way as described during the minimization stage of the protein. The temperature of the protein and the solvent was maintained separately by using velocity rescaling with a stochastic term 62. The coupling time was set to 0.1 ps. The last MD equilibration step was performed with constant pressure and temperature (NPT ensemble) using the canonical sampling through velocity rescaling 62 and the Parrinello–Rahman barostat 63, 64 to maintain the temperature and pressure, respectively. Isotropic pressure coupling with a time constant of 1 ps was used to maintain the pressure at 1 bar. The compressibility was set at 4.5 × 10^−5^ bar^−1^. All of the NVT equilibration steps were carried out for 100 ps each and the NPT equilibration step was carried out for 200 ps.

In all MD simulations, an integration step of 2 fs was used. A cutoff radius of 1.4 nm was employed for the evaluation of both the van der Waals and short‐range electrostatic interactions. Long‐range electrostatic interactions were treated with a particle‐mesh Ewald summation 65 using cubic interpolation with a maximum grid spacing of 0.135 nm for the fast Fourier transform. The neighbor list within the radius of 1.4 nm was updated every 20 fs. The LINCS algorithm 66 was used to constrain bond lengths for the protein using fourth order expansion and two iterations. The SETTLE algorithm 67 was used to constrain water molecules.

We simulated a total of 60 ns for the monomers, 100 ns for each of the unfolded αS dimer model systems, and 60 ns for the tetramer. The corresponding RMSD plots are shown in Fig. S1. The resulting MD trajectories were used to analyze the structural changes and the folding process of αS, as well as for the analyses using the essential collective dynamics (ECD) methodology.

### Essential collective dynamics analysis

We analyzed the αS model systems using the novel essential collective dynamics method (ECD) 44, 46. The theory and derivation of the method have been published previously 44. In brief, the essential collective dynamics is an approach for the analysis of the slow motions of large molecules such as proteins allowing for the probing of persistent dynamics correlations from a relatively short MD trajectory of the molecule. The method is based on a statistical‐mechanical framework in which a macromolecule can be described by a set of generalized Langevin equations (GLEs) employing principal eigenvectors derived through principal component analysis (PCA) of MD trajectories as essential coordinates. The time evolution of a system of N atoms is represented as a time series in 3N‐dimensional configuration space, $\vec{X}\left(t\right)=X_{1}\left(t\right),X_{2}\left(t\right),\ldots ,X_{3N}\left(t\right),$ and PCA is applied to the MD trajectories via the construction of a symmetric covariance matrix *C*_*ij*_,

(1) $$C_{ij}=\left\langle \left(X_{i}\left(t\right)-\left\langle X_{i}\right\rangle \right)\left(X_{j}\left(t\right)-\left\langle X_{j}\right\rangle \right)\right\rangle ,\;i,j=1,2,\ldots ,3N$$

for a subnanosecond segment of the trajectory. The eigenvectors $\vec{E^{k}}$ of the covariance matrix are then arranged according to the corresponding eigenvalues:

(2) $$\vec{E^{k}}=\left\{E_{1}^{k},E_{2}^{k},\ldots ,E_{3N}^{k}\right\},\;k=1,2,\ldots ,3N.$$

By applying PCA on a subnanosecond segment of an equilibrated MD trajectory, the first *k*_*max*_ principal components are identified. Around 10–30 principal components usually sample 90–95% of the total displacement or more. In the ECD method, a projected all‐atom image of the molecule ${\vec{r}}_{i}$ is constructed in the space of the first *k*_max_ principal components 44, 46,

(3) $$d_{ij}=\left|{\vec{r}}_{i}-{\vec{r}}_{j}\right|$$

where $\vec{r_{i}}{}^{k}=\left\{E_{i,x}^{k},E_{i,y}^{k},E_{i,z}^{k}\right\}$ represent triplets of directional cosines of principal eigenvector ${\vec{E}}^{k}$ relative to *x*,* y*,* z* degrees of freedom of atom *i* in the configuration space. Based on an analysis of the GLEs it has been shown 44 that distances between images of atoms, $d_{ij}=\left|{\vec{r}}_{i}-{\vec{r}}_{j}\right|$, represent the level of correlation of atoms *i* and *j* regardless of their proximity in the primary, secondary, or tertiary structure of the protein. It was also demonstrated 49 that the distances *d*_*ij*_ represent persistent correlation in a molecule allowing extrapolating the predictions beyond the fragments of MD trajectories employed for the analysis. The ECD method employs several descriptors based on the distances *d*_*ij*_. They include the dynamic domains of correlated motions 44, the main‐chain flexibility profiles of the protein 45, and the pair correlation maps for main‐chain and side‐chain atoms 50. These descriptors are directly comparable with NMR structural data obtained on a longer time scale than required for the ECD analysis 44, 45, 48, 49, 50.

In this work we employ local main‐chain flexibility, $F_{C_{\alpha }}$, determined as a distance between the images of the *C*_α_ atoms and the centroid which is calculated over the coordinates of all *C*_α_ atoms:

(4) $$F_{C_{\alpha }}\left(i\right)=\left|{\vec{r}}_{i}^{C_{\alpha }}-\vec{\varepsilon }\right|,\;\text{where}\;\vec{\varepsilon }=\frac{1}{N_{C_{\alpha }}}\sum {\vec{r}}_{i}^{C_{\alpha }}.$$

$F_{C_{\alpha }}$ *(i)* represents the level of dynamic coupling of the motion of *i*th *C*_α_ atom with respect to the average motion of the entire main chain of the protein. A low value of $F_{C_{\alpha }}$, that is, low flexibility, represents a strong correlation with the motion of the entire main chain. In globular proteins with developed secondary structure, such as prion proteins, stable α‐helices or β‐strands are often found in regions of low flexibility. A high value of $F_{C_{\alpha }}$, identifies more flexible parts of the protein 45, 48, 50.

We also use pair correlation descriptor, *d*_*ij*_, obtained directly from the distance between images of atoms *i* and *j* in the projected image ${\vec{r}}_{i}$,

(5) $$d_{ij}=\left|{\vec{r}}_{i}-{\vec{r}}_{j}\right|.$$

The descriptor *d*_*ij*_ is a dimensionless quantity such that a low value of *d*_*ij*_ means that the atoms are moving coherently (i.e., strong correlation), whereas larger *d*_*ij*_ values indicate that the motion is less correlated. The pair correlation descriptors are typically visualized in the form of correlation maps 50.

We have analyzed each αS model system using the last 10 ns of the corresponding 60‐ or 100‐ns MD trajectories. Local main‐chain flexibilities 45 were determined for the monomer, dimer, and tetramer models. Pair correlation plots were also constructed for C_α_ atoms of the αS monomer, dimer, and tetramer molecules. Average flexibility and average pair correlations for each model system were computed using fifty 0.2‐ns‐long segments from the last 10 ns of the 60‐ or 100‐ns MD trajectory.

## Results

We studied the behavior of initially unfolded monomeric, dimeric, and tetrameric forms of αS in water during the course of MD simulations. The starting configurations of the unfolded monomer, the dimer, and the tetramer used in this study are shown in Fig. 2. The dimers are modeled in two configurations, parallel and antiparallel, which are labeled as either ‘head‐to‐head’ (HH) or ‘head‐to‐tail’ (HT). In the dimer configurations, the difference between configurations #1 and #2 is that in #2, the second monomeric chain is rotated by 180° along the main‐chain axis, and therefore the initial orientation of side chains is different. The tetramer configuration was made by two units of the HT2 dimer such that there is an equidistant separation among the monomeric units. Initial chain separation of 14 Å was used. To facilitate the identification of the protein terminal tails, the N‐terminal and C‐terminal tails are indicated by blue and red spheres, respectively.

We performed the MD simulations by starting from the unfolded structures of αS without any of the secondary structure elements as shown in Fig. 2. The simulations lasted for 60 ns for most cases, and for 100 ns for several dimer structures, at the temperature of 310 K. Snapshots of the MD trajectories illustrating the folding process, timelines for the evolution of the secondary structures, and details of the analyses using the ECD method are discussed below. Other relevant results are given in the Supporting Information.

### Monomer

**Figure 3:**
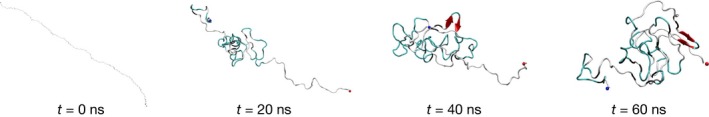
Secondary structure evolution of the monomer in the course of a 60‐ns simulation. The snapshots of the structure at *t* = 0, 20, 40, and 60 ns are shown.

We performed three independent 60‐ns production MD simulations for the monomer. Snapshots of the structures at time 0, 20, 40, and 60 ns for one of the production MD simulations are shown in Fig. 3, and the evolution of the secondary structures is depicted in Fig. S2. Early in the simulations, the monomer collapses to form random coils without a stable secondary structure. As the simulation progresses, we can see the appearance of β‐sheets and isolated β‐bridges in various parts of the protein chain. Over the first 20 ns, the β‐structures are seen appearing and disappearing in a transient manner in the course of the simulation. Toward the last 20 ns of the trajectory, a stable β‐sheet has formed where β‐bridges have been often found earlier. One α‐helix, and a 3_10_‐helix structure have been appearing occasionally. Table 1 shows the number and the corresponding identity of residues of all β‐sheets, β‐bridges, α‐, and 3_10_‐helices identified over the last 10 ns in this MD trajectory. Residue sequences populated by stable β‐sheets for more than 80% of the time are highlighted with a sign (*). One stable antiparallel β‐sheet formed by residues 82–83 and 86–87 has been identified in the monomer. In general, our data indicate that the monomer folds into an irregular conformation dominated by random coils with many isolated β‐bridges. β‐sheets, α‐helices, and 3_10_‐helices also appear occasionally. The locations of the secondary structures, however, were found in different regions in the protein chain across different MD trajectories performed. The observed folding process of the monomer is consistent with the behavior of an intrinsically disordered protein in solution 22, 23.

**Table 1:** Positions and names of residues and the locations of all β‐sheets^a^, β‐bridges, α‐, and 3_10_‐helices identified for the αS monomer over the last 10 ns of the MD trajectory illustrated by Fig. 3

β‐sheets	*82VE83	*86GS87	105EG106											
β‐bridges	M5	G7	K10	A11	K12	V16	A18	A19	T22	V26	A29	T33	V37	E46
	V49	H50	T54	A56	T59	T64	N65	G67	G68	V71	K80	V82	E83	G84
	G86	S87	T92	G93	V95	K96	G101	E104	E105	G106	E110	P117	P120	E139
α‐helix	45KEGV48													
3_10_‐helix	57EKT59													

a β‐sheets that are observed for more than 80% of the time are highlighted with a (*).

**Figure 4:**
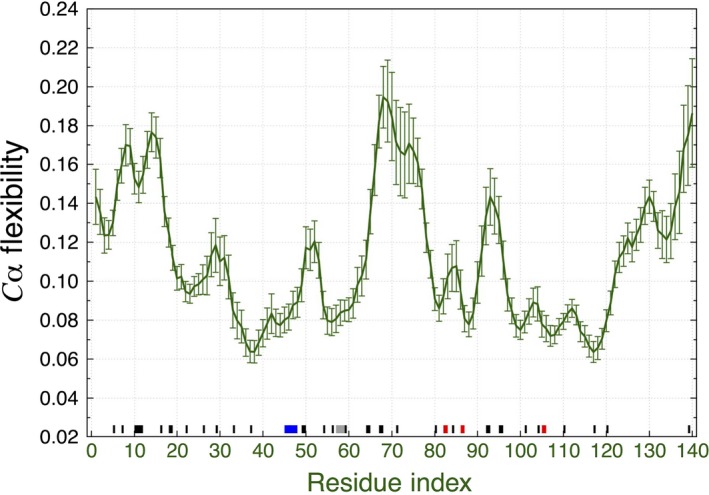
Average main‐chain ECD flexibility profile of the monomer. The locations where secondary structures were identified according to Table 1 are shown with black‐, red‐, blue‐, and gray‐colored bars for isolated β‐bridge, β‐sheets, α‐helix, and 3_10_‐helix, respectively, along the *x*‐axis corresponding to the residue number.

To analyze the folding dynamics of the monomer into a greater depth, we applied the ECD method onto the last 10 ns of the MD trajectory. We analyzed fifty 200‐ps segments. Each MD segment contained 2000 protein conformations excluding the water molecules and the ions. PCA was applied using 20 principal components for each MD segment and taking into account all heavy atoms, that is, excluding hydrogen atoms of the protein. We then computed the average main‐chain ECD flexibility profile for the monomer from Fig. 3 [see Eqn (4) in the Materials and methods section]. In Fig. 4, a sequence of maxima and minima of the flexibility is visible at random locations. In the graph, low values of the flexibility descriptor indicate relatively rigid locations in the chain, whereas high values of the flexibility descriptor represent more mobile parts. It can be seen that two stable β‐strands, 82–83 and 86–87, are located close to the local minima of the flexibility. However, no pronounced connection between the locations of other flexibility minima and secondary structure elements has been established due to the transient nature of the majority of secondary structure.

**Figure 5:**
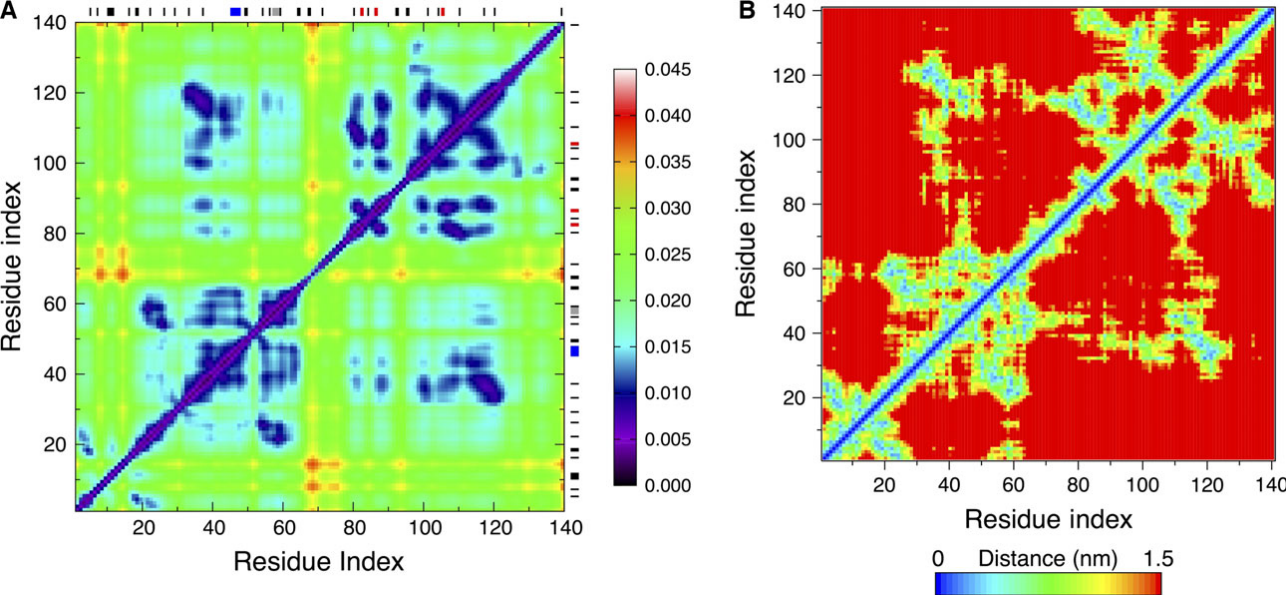
(A) Average C_α_‐atom correlation plot of the monomer calculated from the last 10 ns of a 60‐ns MD trajectory. Low values of the descriptor *d*_*ij*_ in the plot (purple and blue colors) correspond to strong correlations. High values of the descriptor *d*_*ij*_ (yellow and red colors) correspond to relatively independent motion. The locations where secondary structures were identified are shown with black‐, red‐, blue‐, and gray‐colored bars for β‐bridges, β‐sheets, α‐helix, and 3_10_‐helix, respectively, opposite the residue numbering axes. (B) Mean shortest distance map for the monomer calculated from the last 10 ns of a 60‐ns MD trajectory.

Next, we have analyzed the pair correlations of the C_α_‐atoms throughout the monomer [see Eqn (5) in the Materials and methods section]. We have computed the average values of the ECD correlation descriptor over multiple short 200‐ps segments and visualized them in the form of a correlation map. Figure 5A shows the average C_α_‐atom correlation plot of the monomer calculated from the last 10 ns of the 60‐ns MD trajectory. Several regions of highly correlated motions can be identified from Fig. 5A. Some of these highly correlated regions involve the residues where β‐strands or β‐bridges are located. For example, motion of the stable β‐sheet involving residues 82–83 and 86–87, shows a pronounced correlation with residues 98–122, where a β‐strand formed from residues 105–106 and several β‐bridges are contained. The mean shortest interatomic distance map for the collapsed monomer is given in Fig. 5B. Comparison of Fig. 5A, B reveals numerous coincidences of strong pair correlations with close interchain contacts. However, not all close contacts result in strong correlations. For example, *C*‐terminus of the monomer exhibits close contacts, but not dynamical correlations, with residues 80–90. Overall, the described analysis indicates that the monomer folds into an irregular conformation dominated mostly by random coils without pronounced secondary structure, although a minor β‐sheet content may appear occasionally. Such β‐sheet content seems to promote the strongest intrachain interactions in the monomer.

### Dimers

**Figure 6:**
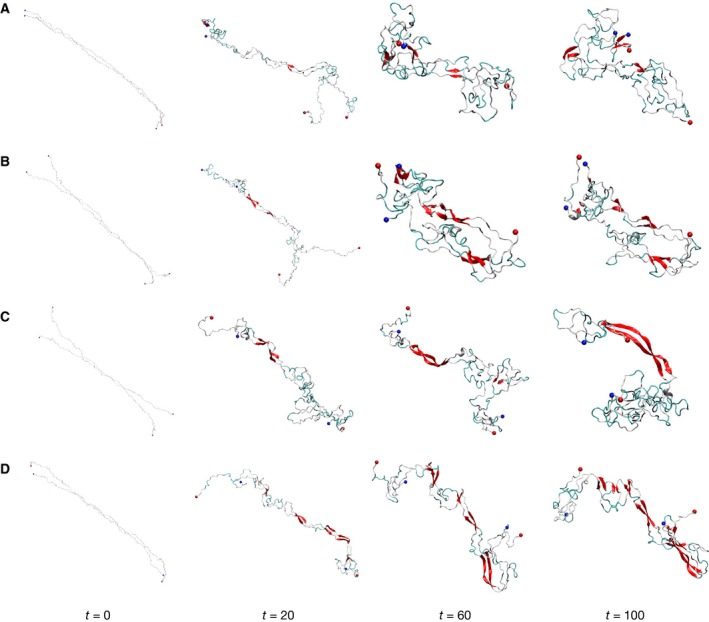
Secondary structure evolution of the dimers in the course of a 100‐ns simulation. The snapshots of the structures illustrated are taken at simulation times *t* = 0, 20, 60, and 100 ns: (A) HH1; (B) HH2; (C) HT1; and (D) HT2.

We performed three independent 100‐ns production MD simulations for head‐to‐head dimers HH1 and HH2 (Fig. 2B, C, respectively) and head‐to‐tail dimers HT1 and HT2 (Fig. 2D, E, respectively) each. At the beginning of the preproduction MD simulations, the dimer model systems were constructed from two unfolded monomeric units of αS (Fig. 2A) and separated by a 14 Å distance. Figure 6A–D displays snapshots of the molecular structures of the HH1, HH2, HT1, and HT2 dimers, respectively, at time 0, 20, 60, and 100 ns. In order to illustrate how the secondary structures evolved in the course of the simulations, typical secondary structure timelines for HH1, HH2, HT1, and HT2 dimers are shown in Figs S3 through S6, respectively.

We have observed that as early as around 2 ns in the simulation of the dimers, β‐strands start forming and remain stable during the course of the simulations. As the simulations progress, more β‐structures are formed. However, the location where those stable β‐structures developed varies with each of the dimer systems. We identified the residues responsible for the formation of these secondary structures. Table 2 lists the positions and names of all residues where β‐strands are observed in each of the dimers between 90 and 100 ns of the respective MD trajectories. As in the case of the monomer, sequences populated by stable β‐sheets for more than 80% of the time are highlighted. Most of the β‐strands identified in Table 2 include one or more of the hydrophobic amino acids F, I, L, M, V, and Y, and polar amino acids Q and T, which are known to be strong β‐sheet formers 68, 69, 70. β‐strands develop in all the three regions of αS chains, as long as at least one of the strong β‐sheet former amino acids is found nearby. β‐strands in the dimers tend to form two‐stranded parallel or antiparallel β‐sheets. Two strands in the β‐sheets belong to different chains at adjacent locations in most cases. The exceptions involve a stable β‐sheet formed by residues 2–6 and 10–13 of chain 2 in dimer HH1, and a stable β‐sheet formed by residues 89–95 and 99–103 of chain 1 in dimer HT1.

**Table 2:** Positions and names of residues where all β‐strands^a^ are observed in each chain of the dimers over the last 10 ns of the corresponding MD trajectories

Dimer	Chain 1 β‐strand	Note	Chain 2 β‐strand	Note
HH1	*35EGVLYV40		*2DVFMK6	
	*64TNVGGAVVT72	NAC	*10KAKE13	
	76AVAQK80	NAC	*37VLYVGS42	
	112ILED115		*69AVVTGVT75	NAC
			79QKT81	NAC
			110EGIL113	
			138PE139	
HH2	6KG7		4FM5	
	16VAAAEK21		7GLSKA11	
	30AGKTK34	RM	39YVG41	
	38LYV40		*44TK45	RM
	*45KE46	RM	*49VHGVATVAEKTKEQV63	RM
	*50HGVATVAEKTKEQV63	RM	*75TAVAQK80	NAC
	*77VAQKTVEG84	NAC	97KDQ99	
			103NEEG106	
			*112IL113	
HT1	7GLS9		8LS9	
	*19AEKTKQGVAEAAGKTKEGV37	RM	13EGVVA17	
	51GV52		32KTKEG36	RM
	*89AAATGFV95	NAC	38LY39	
	*99QLGKN103		72TG73	
	128PS129		*90AATGFVKKDQLGKNEEGAP108	NAC
			113LEDM116	
HT2	28EAAG31		6KG7	
	*35EGVLY39		*20EK21	
	44TK45	RM	*28EAAGKTKEGVLYVGSKT44	RM
	*56AEKT59		*49VHGVATVAE57	
	*66VGGAVVTG73	NAC	*61EQVT64	NAC
	*81TVEGAGSIAAATGFVK96	NAC	68GA69	NAC
	*117PVD119		75TA76	NAC
	127MP128		82VE83	NAC
			*88IAAATGFV95	NAC

a The most stable β‐sheets that are observed for more than 80% of the time are highlighted with a (*). Overlaps with repeat motifs (RM) and (NAC) residues are indicated.

Experiments suggest an association of αS multimerization with the conserved repeat motifs KTK(E/Q)GV 28. Consistent with this, it is evident from Table 2 that the residues of the conserved hexamer motif repeat are frequently involved in the formation of β‐strands in the dimers. Also, many β‐strands are found in the central region of αS, or the NAC domain, which is known to play an important role in the oligomerization of αS 13, 14, 15, 16. In particular, stable β‐strands spanning through the central region of the NAC domain (residues 68–82) are observed in three of the four dimers. Amino acids from peripheral regions of the NAC domain are also involved in the formation of stable β‐strands, particularly in dimers HT1 and HT2. Regions adjacent to the NAC domain are also frequently involved. In HT1, chain 1 has developed a stable antiparallel, intrachain β‐sheet formed from residues 89–95 and 99–103. Notably, the monomer (Table 1) also exhibits a stable antiparallel β‐sheet in a nearly located region. Alternatively, β‐content that does not involve the repeat motifs or residues from the NAC region, tends to be located in N‐terminal regions of the chains.

The formation of α‐, π‐, and 3_10_‐helices was also observed during the simulations as Table 3 indicates. These helices, however, tend to appear and disappear at various points in the trajectories, remaining for < 50% of the time. The amino acids A, E, H, and V, which are found frequently in helical regions 68, are often involved in the formation of the transient α‐ and 3_10_‐helices, as Table 3 shows.

**Table 3:** Positions and names of all residues where α‐ and 3_10_‐helices are observed in the dimers during the last 10 ns of the trajectories

Dimer	α‐helix	3_10_‐helix
HH1	None observed	Chain 1: 16VAA18 47GVV49 133YQD135
		Chain 2: 75TAV77 132GYQ134
HH2^a^	Chain 1: 82VEGA85 87SIAAA91	Chain 1: 3VFM5 15VVA17 123EAY125
	Chain 2: None observed	Chain 2: 12KEGV15 82VEGA85 88IAAA91
HT1	Chain 1: None observed	Chain 1: None observed
	Chain 2: 3VFMK6 53ATVA56	Chain 2: 3VFMK6 12KEG14 47GVVH50 52VATV55 87SIA89
HT2	Chain 1: 49VHGV52	Chain 1: 48VVH50 76AVAQK80
	Chain 2: None observed	Chain 2: None observed

a An α_π_‐helix was also observed in chain 1 of HH2 dimer (residues 83–87, EGAGS).

**Figure 7:**
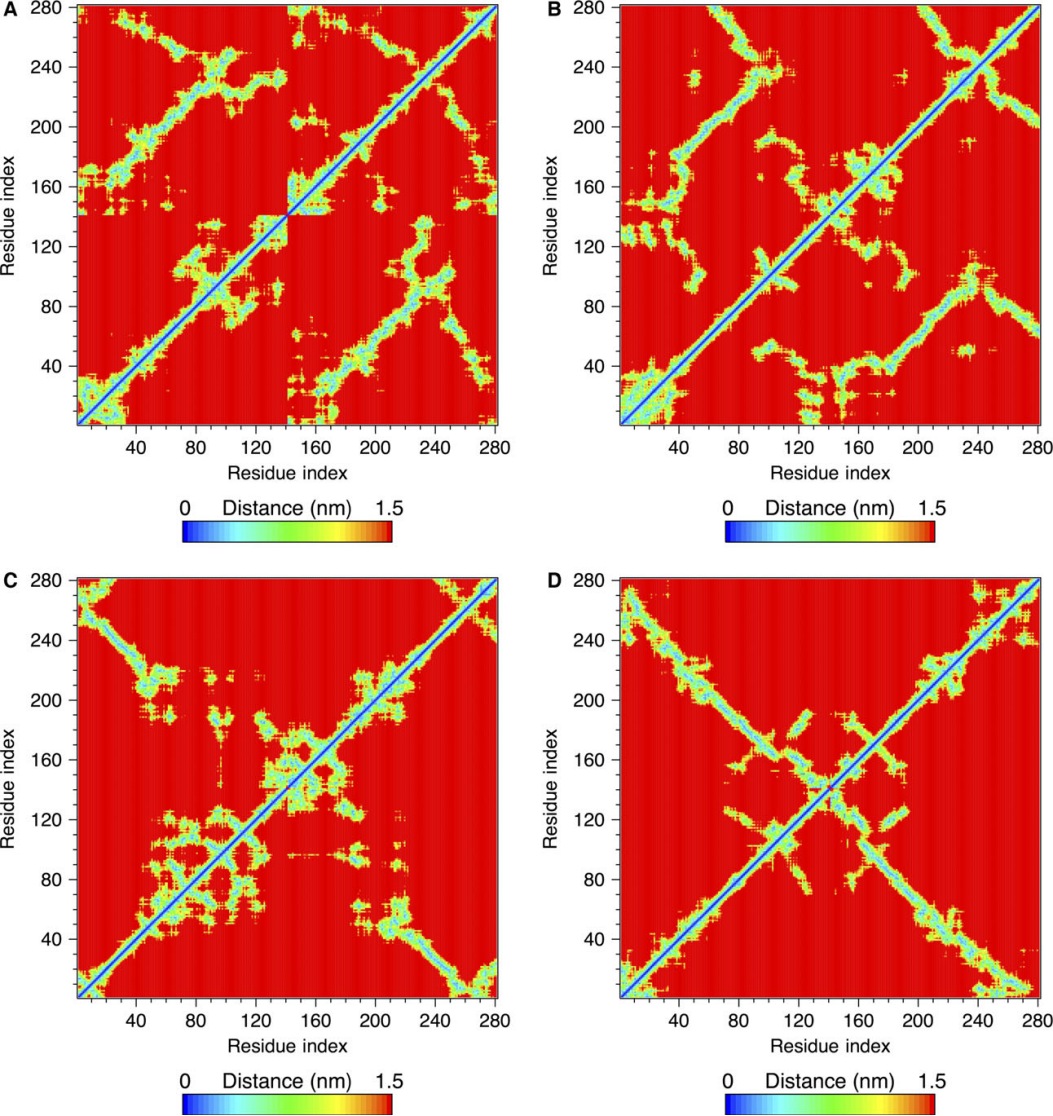
Mean shortest distance maps for the dimers calculated from 90 to 100‐ns MD trajectory: (A) HH1; (B) HH2; (C) HT1; (D) HT2.

Figure 7 shows the interatomic distance maps for the dimers listed in Table 2. It is clearly seen that in each of the two HH dimers (Fig. 7A,B) many interchain contacts are formed. The majority of the closest contacts occur near the central regions of both chains where stable β‐strands can also be found forming parallel β‐sheets. In both dimers, the central parts have retained their head‐to‐head alignment, whereas a significant part of C‐terminal regions has unbuckled and adopted an antiparallel HT‐like alignment with adjacent regions of the dimer. Antiparallel β‐sheets have been observed in these regions.

Comparison of the distance maps for two HT dimers indicates that their behavior is somewhat different from each other. In HT1 (Fig. 7C), residues near N‐terminal tail of chain 1 have remained in a close contact with residues near the C‐terminal of chain 2. Interestingly, this region also has developed a long two‐stranded antiparallel β‐sheet consisting of residues 19–37 from chain 1 and residues 90–108 from chain 2. However, most of the other parts of the dimer have separated and formed mainly random coils with little stable secondary structure. In HT2 (Fig. 7D), the two chains have retained most of their head‐to‐tail alignment, and stable β‐strands have formed in various locations of each chain. β‐strands from one chain form antiparallel β‐sheets with the adjacent β‐strands of the other chain in most cases, such as for example, a β‐sheet formed from residues 81–96 of chain 1 and residues 28–44 of chain 2. The formation of such a long and stable β‐strand close to the C‐terminal region of the NAC domain in one chain seems to be influenced by the presence of a complementary antiparallel β‐strand of similar length in the N‐terminal region of the other chain. This is clearly in contrast with the case of the αS monomer, where stable β‐strands rarely form in this region in the absence of a proper chain alignment (see Table 1).

**Figure 8:**
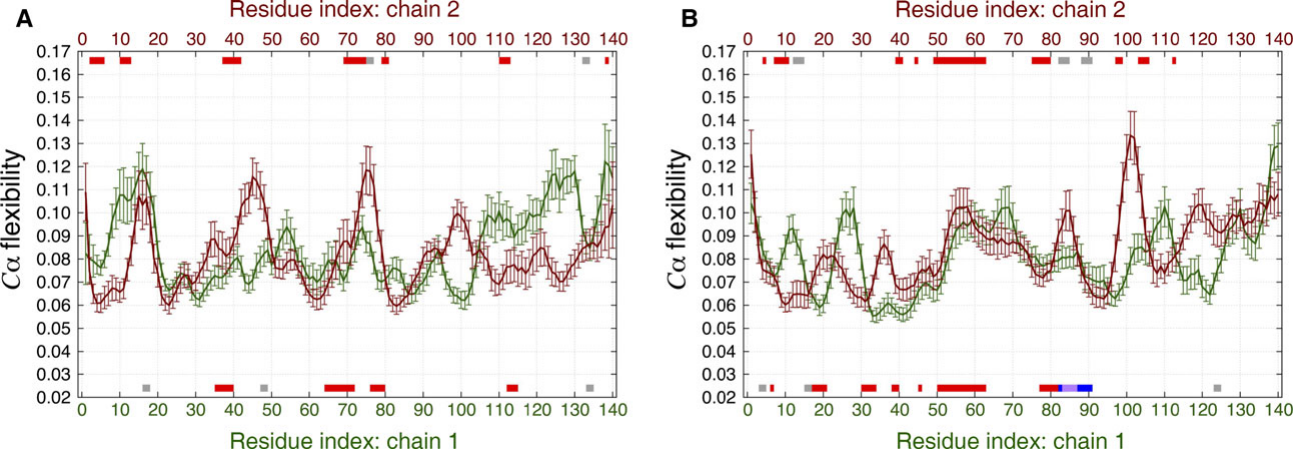
Average main‐chain ECD flexibility profiles of the (A) HH1 and (B) HH2 dimers. The locations where secondary structures were identified are shown with red‐, blue‐, purple‐, and gray‐colored bars for β‐sheets, α‐helix, π‐helix, and 3_10_‐helix, respectively, along the *x*‐axes corresponding to the residue numbers of chain 1 (green) and chain 2 (dark red).

**Figure 9:**
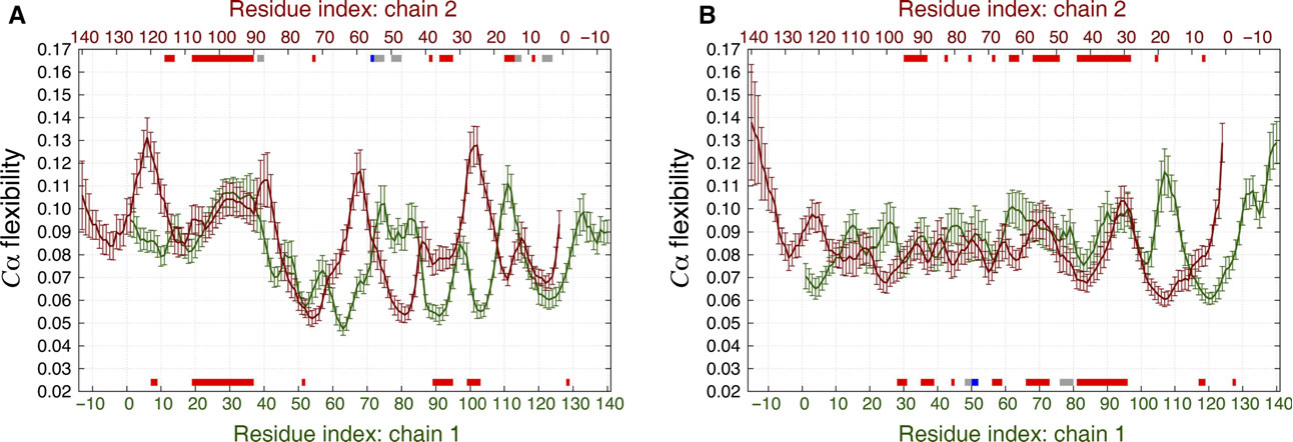
Average main‐chain ECD flexibility profiles of the (A) HT1 and (B) HT2 dimers. The locations where secondary structures were identified are shown with red‐, blue‐, and gray‐colored bars for β‐sheets, α‐helix, and 3_10_‐helix, respectively, along the *x*‐axes corresponding to the residue numbers of chain 1 (green) and chain 2 (dark red).

To quantitatively compare the dynamics of the dimer structures, we performed ECD analysis for each dimer using the last 10 ns of the simulations between 90 and 100 ns of the MD trajectories. The average main‐chain ECD flexibility profiles for the HH and HT dimers are shown in Figs 8 and 9, respectively. All residues involved in the formation of secondary structure elements, as listed in Tables 2 and 3, are indicated by color bars along the top and bottom of the *x*‐axes. In most cases, the alignment of β‐strands across the two chains of each dimer can be clearly seen. It is also evident that the nature and positions of the secondary structure elements, as well as the shapes of the flexibility profiles displayed in Figs 8 and 9 are different from the corresponding plot for the monomer in Fig. 4.

In globular proteins, regions of ECD flexibility profiles with relatively high values usually correspond to random coils and loops, and regions of minima typically indicate the location of stable α‐helices and β‐sheets 45, 48, 49, 50, 51, 52, 53. However, the stable β‐sheets which developed during the MD simulations of the αS dimers seem to influence the flexibility in a somewhat different way. In the case of the HH dimers in Fig. 8, the dynamics of N‐terminal and central regions in one chain seems to be influenced by adjacent regions in the other one. When an interchain, two‐stranded β‐sheet develops, this seems to result in a certain degree of alignment of the main‐chain flexibility profiles of the two chains. Such an alignment can be seen in Fig. 8A, particularly for residues 30–40 and 60–75, and in Fig. 8B for residues 40–80. This involves the alignment of the levels of the flexibility, as well as close positions of maxima and minima across two chains in these regions. However, stable β‐sheets do not necessarily coincide with minima of the main‐chain flexibility in these dimers. For example, the longest β‐sheet in HH2 is located close to local maxima of the flexibility profiles in both chains.

The ECD flexibility profiles for the HT dimers are depicted in Fig. 9. The flexibility profiles of the second chain were flipped relative to the first chains and also shifted by a certain number of residues depending on which residues in the two chains are in close proximity. This flipping of the second chain was done in accordance with the 3D structure geometry of each of the two dimers. Similarly to the HH dimers, the dynamics of one chain seems to be influenced directly by the other one in the regions where stable interchain β‐sheets are observed. The maxima and minima of the flexibility profiles of the two chains are found at close locations, and the levels of the flexibility adopt close values in these regions. In HT1 dimer (Fig. 9A), such an alignment of the dynamics is found in the region of β‐sheet involving residues 19–37 of chain 1 and residues 90–108 of chain 2. In HT2 dimer (Fig. 9B), adjacent chains exhibit close levels of flexibility throughout an extensive central region, where multiple stable β‐sheets are observed.

**Figure 10:**
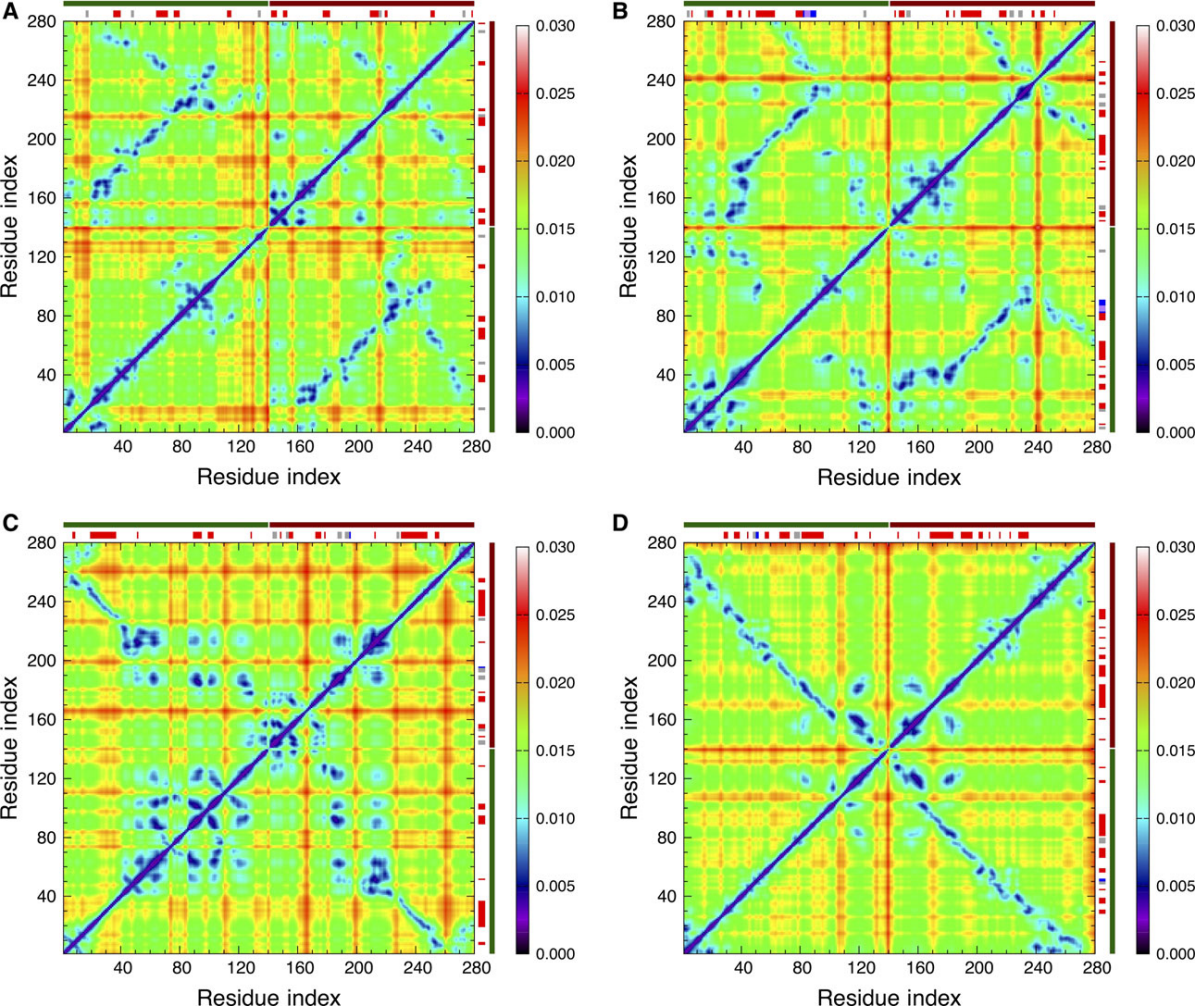
Average C_α_‐atom correlation plot of the dimers calculated from the last 10 ns of a 100‐ns MD trajectory: (A) HH1; (B) HH2; (C) HT1; and (D) HT2. Low values of the descriptor *d*_*ij*_ in the plot correspond to strong correlations and high values correspond to a relatively independent motion. The color scheme is as in Fig. 5A. The locations where secondary structures were identified are shown with red‐, blue‐, purple‐, and gray‐colored bars for β‐sheets, α‐helix, π‐helix, and 3_10_‐helix, respectively, opposite the residue axes. The longer dark green and dark red bars indicate chains 1 and 2, respectively.

Next, we have calculated the ECD pair correlations of the C_α_‐atoms for all the dimers and visualized them in the form of correlation maps. As in the case of the monomer, we performed the calculations of the average values over multiple short 200‐ps segments using the last 10 ns of the 100‐ns MD trajectory. The average C_α_‐atom correlation maps for the HH and HT dimers are shown in Fig. 10.

Regions of highly correlated motions can be identified from the correlation maps. In Fig. 10A, B, the correlations observed in HH1 do not differ significantly from HH2. Similar regions can be identified in both plots, indicating that similar residues participate in correlated motions regardless of the different orientation of the side chains against each other in the initial constructs. Regions of the highest interchain correlations shown in blue color in Fig. 10A, B follow closely the contact distance maps in Fig. 7A, B, often involving stable interchain β‐sheets. However, major β‐strands tend to show strong interchain correlations almost exclusively within their β‐sheets. Correlations of stable β‐strands with other regions tend to be relatively weak. Consistent with the contact distance maps in Fig. 7A, B, it is also evident that in both HH dimers, the C‐terminal tail in one of the chains has folded back and developed correlations representative of a partially HT‐like orientation.

Unlike the HH dimers, pair correlations plots of the HT1 and HT2 dimers are pronouncedly different, as Fig. 10C, D, respectively, illustrates. As Figs 6C and 7C indicate, the two chains of HT1 dimer have largely separated, whereas in HT2 dimer the two chains have retained their alignment. Nonetheless, in both HT dimers, stable β‐structures as well as regions of highly correlated motions are observed. For HT1 dimer, the correlation map shown in Fig. 10C exhibits a strong correlation within the longest antiparallel β‐sheet consisting of residues 19–37 of chain 1 and residues 90–108 of the complementary β‐strand in chain 2. However, beyond the long β‐sheet and adjacent regions, the chains of HT1 have separated and collapsed into globular‐like structures with many random coils. Correlations of the collapsed regions exhibit maxima at random locations, reminiscent of the dynamics of the monomer. In HT2 dimer (Fig. 10D) highly correlated motions show a pronounced association with pair correlations of stable β‐strands from one chain with complementary β‐strands from the other chain forming β‐sheets. For example, the longest β‐strand identified in HT2 dimer comprising residues 81–96 of chain 1 is strongly correlated with residues 28–44 of chain 2, and similar correlations are observed for other major β‐strands in HT2.

### Tetramer

Next, we have investigated the structure and dynamics of αS tetramer model constructed from two HT2 dimers, as Fig. 2F shows. Although several tetrameric configurations of αS might be constructed, in this study we have focused on the secondary structure stability in a tetramer that involves both parallel and antiparallel configurations of the chains. The tetramer that we have considered can be seen as a combination of two HH dimers and two HT dimers at the same time, all chains interacting with each other as depicted in Fig. 2F. To clarify the interpretation of the interactions among the four chains of the tetramer, Table 4 lists the six pairs of the monomeric chains present in the tetramer, and identifies them as either HH or HT dimeric configurations. The tetramer was constructed such that the separation among the four constituent monomeric chains was similar as in the dimers.

**Table 4:** Six pairs of chains in the tetramer and the resulting dimer configurations

Tetramer chain index	Dimer configuration
1 and 2	HT
1 and 3	HH
1 and 4	HT
2 and 3	HT
2 and 4	HH
3 and 4	HT

**Figure 11:**
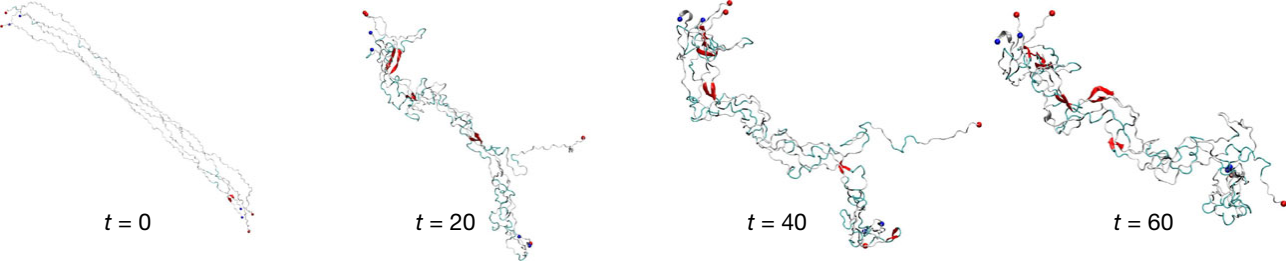
Secondary structure evolution of the tetramer in the course of a 60‐ns simulation showing snapshots of the structure at times *t* = 0, 20, 40, and 60 ns.

We performed three independent 60‐ns production MD simulations on the tetramer model with varying equidistant separations among the monomeric units. The results discussed in this section are for tetramers with a 14‐Å separation among the monomeric chains. From the trajectories of the production MD, we captured snapshots of the tetramer molecular structures at time 0, 20, 40, and 60 ns as shown in Fig. 11. The typical secondary structure timelines for this tetramer are displayed in Fig. S7. Early in the production simulation, β‐strands started forming in the tetramer. As the simulation has progressed, more secondary structures have formed including α‐ and 3_10_‐helices (see Fig. S7). Table 5 lists the positions and names of residues where all β‐sheets, α‐, and 3_10_‐helices are observed in the tetramer between 50 and 60 ns of the MD trajectory. Residue sequences populated by stable β‐sheets for more than 80% of the time during the last 10 ns of the simulations are marked with an (*). Out of these, two antiparallel β‐sheets and one parallel β‐sheet are present almost 100% of the time in this simulation. These include one antiparallel β‐sheet formed by residues 15–16 of chain 1 and residues 15–17 of chain 3; and another antiparallel β‐sheet formed by residues 19–23 of chain 1 and residues 98–103 of chain 4. The stable parallel β‐sheet in the tetramer is formed by residues 38–40 of both chains 1 and 3. Two less stable parallel β‐sheets have been also identified in the tetramer. One of these β‐sheets, formed by residues 63–66 of chain 2 and residues 65–68 of chain 4, is present for about 65% of the time in the last 10 ns of the simulation. The other parallel β‐sheet, present for about 25% of the time, is formed by residues 45–53 of chain 1 and residues 46–52 of chain 3. The other β‐sheets listed in Table 5 are even less stable and tend to appear and disappear in a highly transient manner during the simulations.

**Table 5:** Positions and names of residues where all β‐sheets^a^, α‐, and 3_10_‐helices^b^ are observed in the tetramer during the last 10 ns of the trajectory illustrated by Fig. 11

Structure	Chain 1	Chain 2	Chain 3	Chain 4
β‐sheet	*15VV16	7GLSK10	*15VVA17	5MKG7
	*19AEKTK23	23KQGVA27	*38LYV40	35EGVLYVG41
	*38LYV40	34KE35	46EGVVHGV52	50HG51
	45KEGVVHGVA53	39YVGSK43	56AE57	65NVGG68
	55VA56	63VTNV66	65NVGG68	75TA76
	63VTNVGGA69	68GA69	76AV77	*98DQLGKN103
	92TG93		95VKK97	
	99QLG101		100LG101	
			112IL113	
			120PD121	
			127MP128	
α‐helix	109QEGI112	**29AAGK32	None observed	None observed
3_10_‐helix	**2DVF4	**19AEKT22	29AAGK32	29AAG31
	69AVV71	29AAG31	122NEA124	45KEG47
		**98DQL100		**77VAQK80

a β‐sheets marked with (*) are observed for more than 80% of the time.

b α‐ and 3_10_‐helices marked with (**) are observed for more than 50% of the time.

Most of β‐strands that we have identified contain at least one of the amino acids such as I, L, M, Q, T, V, or Y, which are known to be strong β‐sheet formers 68, 69, 70. Parts of the conserved hexamer motif KTK(E/Q)GV were observed in stable β‐sheet content of chain 1 of the tetramer. Several β‐strands coincided with the NAC domain. In particular, residues 63–66 of chain 2 and residues 65–68 of chain 4 have been identified as parts of β‐strands, both of which are present about 65% of the time. Chains 1 and 3 also exhibit β‐strands in regions 63–69 and 65–68, respectively; however, these β‐strands are present only 1% of the time.

We have also identified several α‐helices and 3_10_‐helices in the tetramer, as Table 5 lists. In distinction from the monomer and dimers where all helical content appears in a highly transient manner, in the tetramer several residue sequences are populated by α‐ and 3_10_‐helices for more than 50% of the time during the last 10 ns of the simulations. Such residue sequences are marked with an (**) in the table. They include one α‐helix formed by residues 29–32 of chain 2, and four 3_10_‐helices formed by residues 2–4 of chain 1, residues 19–22 and 98–100 of chain 2, and residues 77–80 of chain 4.

**Figure 12:**
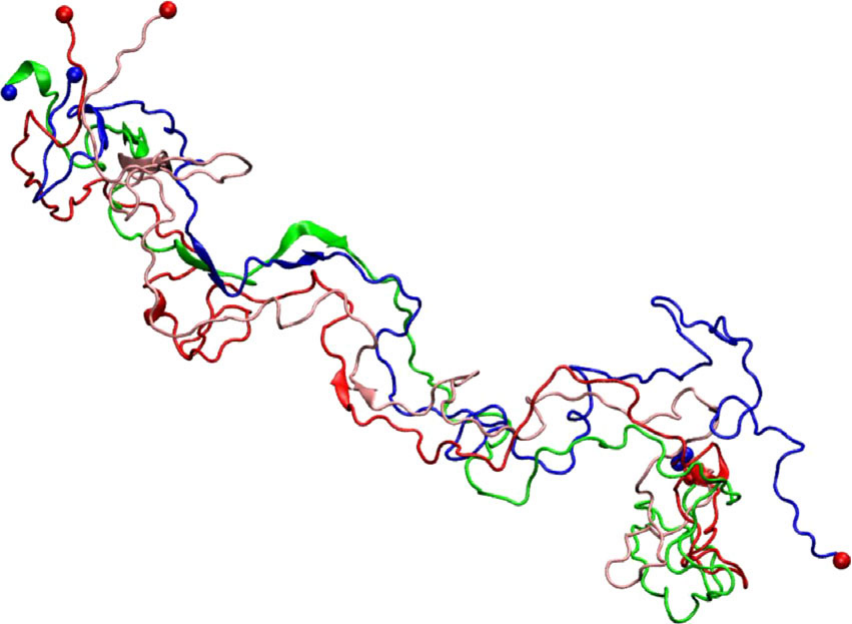
Snapshot of the structure of the tetramer at time *t* = 60 ns. The colors green, dark red, blue, and pink identify chains 1, 2, 3, and 4, respectively.

Figure 12 shows the snapshot of the structure of the tetramer at 60 ns similar to that in Fig. 11, with chains 1, 2, 3, and 4 colored green, dark red, blue, and pink, respectively, to clarify the conformations adopted by each chain. Despite a partial collapse seen in Figs 11 and 12, the tetramer has largely retained the chain alignment. Except for a C‐terminal part of chain 3, most of the interchain interactions have persisted throughout the simulations. In comparison to the dimers, the tetramer shows significantly less stable β‐sheet content. However, the helical content is found to be more stable in the tetramer than in the dimers. These observations appear consistent with experimental studies 25, 26 suggesting that αS may adopt its soluble tetrameric form enriched in helical content in the absence of lipid bilayers or micelles. Although the hypothetical model of helically folded αS tetramer 26 differs from the initial structure studied in this paper, our results indicate that a bundle of four αS chains may promote the presence of helical content, in agreement with experimental observations.

**Figure 13:**
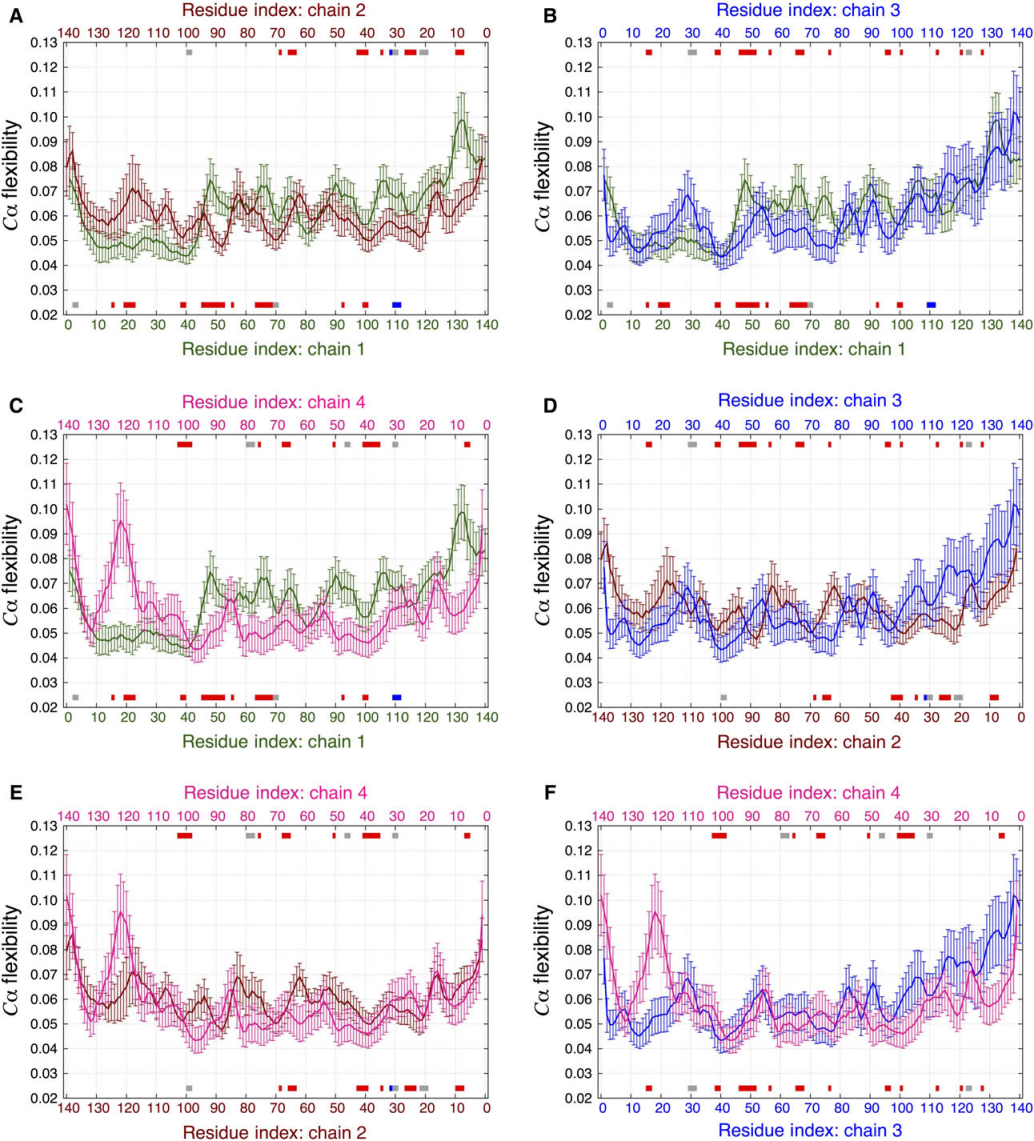
Average main‐chain ECD flexibility profiles of the tetramers plotted as dimer pairs: (A) Chains 1 and 2 (HT); (B) Chains 1 and 3 (HH); (C) Chains 1 and 4 (HT); (D) Chains 2 and 3 (HT); (E) Chains 2 and 4 (HH); (F) Chains 3 and 4 (HT). The locations where secondary structures were identified are shown with red‐, blue‐, and gray‐colored bars for β‐sheets, α‐helix, and 3_10_‐helix, respectively, along the *x*‐axes corresponding to the residue numbers of chain 1 (green), chain 2 (dark red), chain 3 (blue), and chain 4 (pink).

To further investigate the folding dynamics of the tetramer, we calculated the average main‐chain ECD flexibility profiles for each of the four chains as depicted in Fig. 13. The flexibility profiles are plotted in pairs as listed in Table 4. Two of the pairs adopt an HH alignment (Fig. 13B, E) and four of them adopt HT alignments (Fig. 13A, C, D, F). The secondary structure elements identified for each chain are indicated along the top and bottom of the *x*‐axes according to Table 5. For all pairs of chains within the tetramer regardless the alignment, the flexibility profiles adopt close levels with the positions of maxima and minima tending to align in many cases. As in the case of the dimers, flexibility of each chain in the tetramer seems to be influenced by neighboring chains, especially in the central regions. However, the alignment of β‐strands across the chains in the tetramer, as well as the influence of the secondary structure on the flexibility, is less pronounced than in the HH and HT dimers, suggesting somewhat weaker interchain interactions in the tetramer.

**Figure 14:**
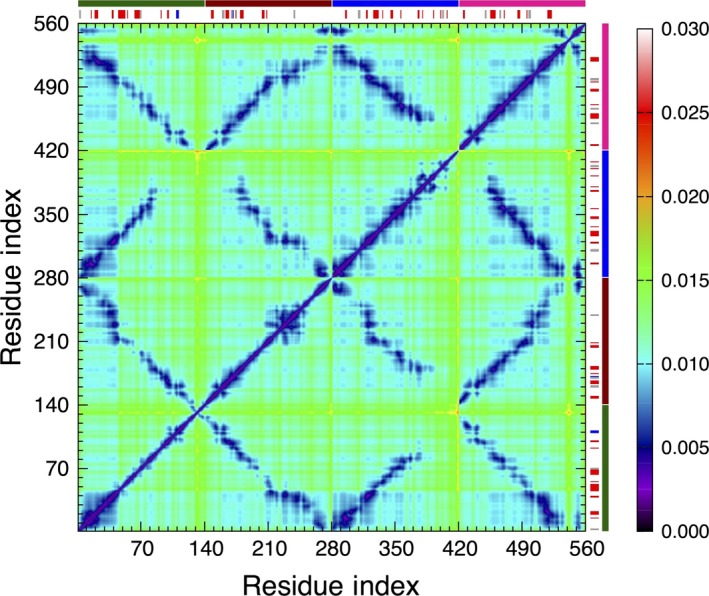
Average *C*_α_‐atom correlation plot of the tetramer calculated from the last 10 ns of a 60‐ns MD trajectory. Low values of the descriptor *d*_*ij*_ in the plot correspond to strong correlations and high values correspond to a relatively independent motion. The locations where secondary structures were identified are shown with red‐, blue‐, and gray‐colored bars for β‐sheets, α‐helices, and 3_10_‐helices, respectively, opposite the residue axes. The longer green, dark red, blue, and pink bars identify the locations of chains 1, 2, 3, and 4, respectively.

The average C_α_‐atom ECD correlation map for the tetramer is shown in Fig. 14. Each quadrant in Fig. 14 represents correlations between a pair of chains, and long color bars identify the chains using a similar color scheme as in Fig. 12. N‐terminal regions of chains 1 and 3 and C‐terminal regions of chains 2 and 4, while maintaining a significant part of their alignment, have collapsed into a globule showing strong interchain correlations throughout the globule. The C‐terminal tail of chain 3 has detached from the rest of the tetramer and therefore lost correlations with the other chains; while the other three chains have retained most of their alignment. In particular, most of the NAC regions of all the four chains have remained well‐aligned and show pronounced correlations between the chains. The most stable β‐sheets identified for the tetramer seem to determine the strongest interchain correlations. For example, residues 15–16 and 38–40 of chain 1 show strong correlated motions with residues 15–17 and 38–40 of chain 3. This is also true for residues 19–23 of chain 1 and residues 98–103 of chain 4. Similarly, the β‐sheets formed between residues 63–66 of chain 2 and residues 65–68 of chain 4, both belonging to the NAC region, also exhibit high levels of correlated motions. The motion of stable α‐helix in chain 2 does not seem to strongly correlate with the other secondary structures. The motion of 3_10_‐helix formed by residues 98–100 of chain 2, however, correlates with the 3_10_‐helix formed by residues 77–80 of chain 4.

## Discussion

We have investigated the folding dynamics of initially unfolded αS chains in water. The model examples considered include a monomer, four dimers comprising two head‐to‐head (HH) and two head‐to‐tail (HT) configurations, and a tetramer. For each model system, we have performed molecular dynamics simulations in water at a temperature of 310 K and monitored the formation and evolution of secondary structure elements such as β‐sheets, β‐bridges, α‐, and 3_10_‐helices. We also have analyzed dynamic correlations in these model systems employing the novel ECD method.

Our results indicate that the initially unfolded αS monomer collapses into an irregular globular conformation dominated by random coils and isolated transient β‐bridges, as expected for an intrinsically disordered protein in solution 22, 23.

In contrast, two hypothetical dimeric structures, HH and HT, exhibit stable two‐stranded parallel or antiparallel β‐sheets, mostly across two chains of the dimers, which are present for more than 80% of the time during the last 10 ns of the simulations. Most of β‐strands identified in the dimers include one or more amino acids that are known to be strong β‐sheet formers. Many of stable β‐strands either are found in the N‐terminal regions of the chains, or are located in the vicinity of conserved hexamer motif KTK(E/Q)GV, or they involve the NAC domain residues. However, the exact positions of these β‐strands are different in the four dimer systems that we have explored. Regions of the dimers where stable interchain β‐sheets have formed retained most of their alignment throughout the simulations. Alternatively, a trend to collapse into irregular structures with many random coils has been observed in parts of the chains without stable β‐content. In both HH dimers, a significant part of C‐terminal regions has unbuckled and adopted an antiparallel HT‐like alignment with adjacent parts of the dimer. More β‐sheet content has been observed in HT dimers than in HH dimers. No stable helical content has been identified in any of the dimers.

Our comparative analysis of structure and dynamics of the hypothetical HH and HT dimeric constructs allows concluding that both parallel and antiparallel alignment of αS chains in water promote a buildup of stable β‐sheets. In order to more closely investigate the relative stability of β‐sheets in the HH and HT alignments, we have analyzed tetrameric constructs composed of two initially unfolded HT dimers, such that the formation of parallel and antiparallel β‐sheets is in competition. In such a tetramer, folding trends different from each of the dimers have been observed. Despite some collapse and occasional buckling of the chains, the tetramer appears to retain the chain alignment and maintain the corresponding interchain interactions better than the dimers. At the same time, the tetramer also exhibits significantly less stable interchain β‐sheets in comparison to the dimers. Most β‐strands detected in the tetramer tend to appear and disappear in a transient manner during the simulations. No pronounced prevalence of parallel β‐sheets over antiparallel ones has been observed. The helical content is found to be more stable in the tetramer than in any of the other constructs considered. We have observed an α‐helix and several 3_10_‐helices present for more than 50% of the time during the last 10 ns of the simulations. Overall, the close contact of two HT dimers appears to decrease their propensity to form stable β‐sheets through competing interactions of the four chains.

To investigate the dynamics of the monomeric, dimeric, and tetrameric models into a greater depth, we have applied the novel essential collective dynamics (ECD) method 44, 45, 46, 47, 48, 49, 50, 51, 52, 53. The method allows for the determination of persistent dynamic correlations of atoms within a protein, and related dynamical determinants such as main‐chain flexibilities, from short MD trajectories. We found that the strongest correlations of motion often occur between pairs of main‐chain atoms located in close proximity, as one could expect. However, the spatial proximity is not the only factor influencing the correlations. For the monomer, some regions of highly correlated motions involve interacting β‐sheets or isolated β‐bridges. In the dimers, high correlations are found between stable β‐strands forming parallel or antiparallel β‐sheets. In such cases, the motion of stable β‐strands in one chain is highly correlated with stable β‐strands in another chain of the dimer. In the tetramer, stable β‐sheets and some of stable 3_10_‐helices also seem to influence interchain correlations of motion. However, due to a lesser number of stable secondary structures in the tetramer, most of the correlations occur just between closely positioned atomic groups. The comparison of ECD flexibility profiles of the monomer, dimer, and tetramer reveals differences in the shape of the profiles. However if one compares the flexibility of two chains in each dimer, the dynamics of one chain seems to be influenced by the other chain in regions where stable interchain β‐sheets are observed. The maxima and minima of the flexibility profiles of the two chains are found at close locations, and the levels of the flexibility adopt close values in these regions. However, stable β‐sheets do not coincide with minima of the main‐chain flexibility in the dimers, distinct from the case of globular proteins 45, 48, 49, 50, 51, 52, 53. For the tetramer, the flexibility of each chain also seems to be influenced by the neighboring chains. However, the influence of the secondary structure on the flexibility profiles is less pronounced in the tetramer than in the dimers.

Overall, our comparative analysis of the folding dynamics of initially unfolded monomeric, dimeric, and tetrameric αS models in water reveals pronounced differences. Although the monomer simply collapses into an irregular globule with transient appearance of secondary structures, both HH and HT dimers develop significant stable β‐sheet content. Interchain β‐sheets, either parallel or antiparallel, seem to play an important role in determining structural stability and dynamics of these constructs. Formation of multiple interchain β‐sheets results is a better retention of the chain alignment. The four chains of the tetrameric construct also have retained their alignment in our simulations. However in distinction of the dimers, structure and dynamics of the tetramer has been found less dependent on the β‐sheet content, which is also less pronounced in the tetramer than in each of the dimers. At the same time, the helical content is found to be more stable in the tetramer than in the other structures, consistent with experimental observations 25, 26.

In agreement with experiments 33, 34, 35, the structure and folding dynamics observed in our dimeric and tetrameric constructs is different from both monomers and mature amyloidal fibrils. The propensities to develop and maintain stable β‐sheet content are clearly more pronounced in the dimers and tetramer than in the monomer. Yet the irregular, random‐coil rich structure of the dimeric and tetrameric models is different from the anticipated morphology of mature fibrils, supporting the hypothesis that major structural conversion of early oligomers is required for the fibrils to be formed 34, 35. Interestingly, both HH and HT constructs appear capable of maintaining their alignment, and they exhibit both parallel and antiparallel β‐sheets. Detailed mechanism resulting in one of the alignments prevailing in amyloidal fibrils has yet to be understood. The details of the structure and folding dynamics of the αS constructs with various hypothetical alignments of the chains described in this work shed light onto the properties of early aggregation intermediates, particularly emphasizing the importance of smaller oligomers, such as dimers, in the process of β‐conversion of α‐synuclein.

## Author contributions

Conceived the study and experiment: MS. Performed the experiments: JM. Analyzed and interpreted the data: JM and MS. Prepared the draft of the article: JM and MS.

## Supporting information


**Fig. S1.** displays the time dependencies of root‐mean‐square deviations (RMSDs) of the monomer (**S1A**), the dimers (**S1B–S1E**), and the tetramer (**S1F**).
**Figs S2–S7.** display the timelines of secondary structure evolution of the monomer (**S2**), dimers (**S3–S6**), and tetramer (**S7**) during MD simulations.
