# Supplementary material for: Understanding the dynamics of monomeric, dimeric, and tetrameric α‐synuclein structures in water
Source: FEBS Open Bio. 2016 Jun 1;6(7):666–86. doi: 10.1002/2211-5463.12069 (PMC4932447; doi:10.1002/2211-5463.12069)
Supplement: Supplementary file 1 — Fig. S1. displays the time dependencies of root‐mean‐square deviations (RMSDs) of the monomer (S1A), the dimers (S1B–S1E), and the tetramer (S1F). Figs S2–S7. display the timelines of secondary structure evolution of the monomer (S2), dimers (S3–S6), and tetramer (S7) during MD simulations. [file FEB4-6-666-s001.docx]

Supporting Information for:

Understanding the Dynamics of Monomeric, Dimeric, and Tetrameric α-Synuclein Structures in Water

Jonathan Y. Mane^1,2^, and Maria Stepanova^1,2,3*^

^1^ Department of Electrical and Computer Engineering, University of Alberta, Edmonton, Alberta T6G 1H9, Canada

^2^ National Institute for Nanotechnology, National Research Council Canada, Edmonton, Alberta T6G 2M9, Canada

^3^ Department of Physics, Astronomy, and Materials Science, Missouri State University, Springfield, Missouri, 65897, United States.

^*^ Corresponding author: [ms1@ualberta.ca](mailto:ms1@ualberta.ca)

**
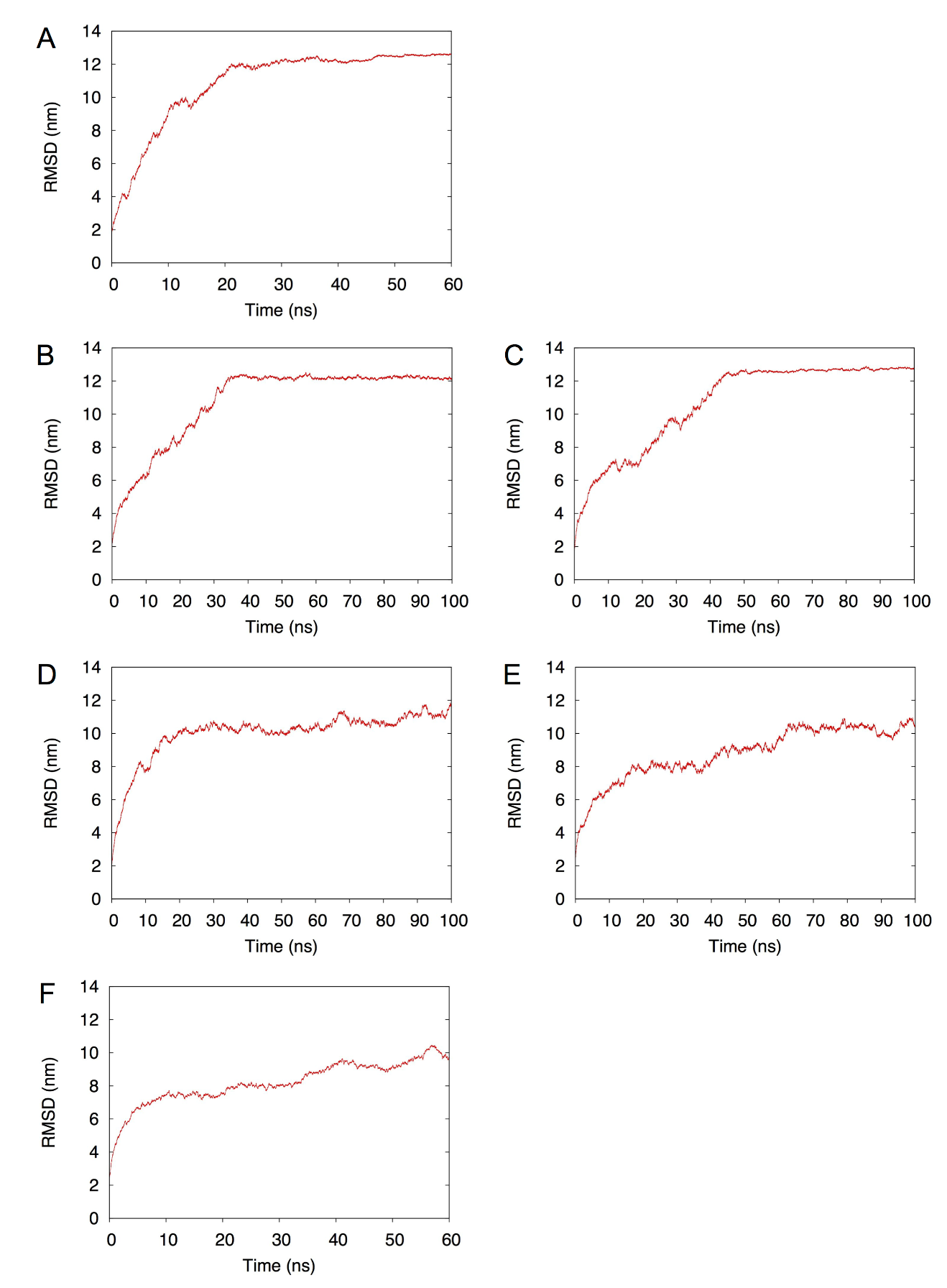
**

**Fig. S1.** Time-dependence plots of the root-mean-square deviations (RMSDs) calculated from the starting conformation of the respective αS models: (A) – monomer; (B) – HH1 dimer; (C) – HH2 dimer; (D) – HT1 dimer; (E) – HT2 dimer; and (F) – tetramer.

**
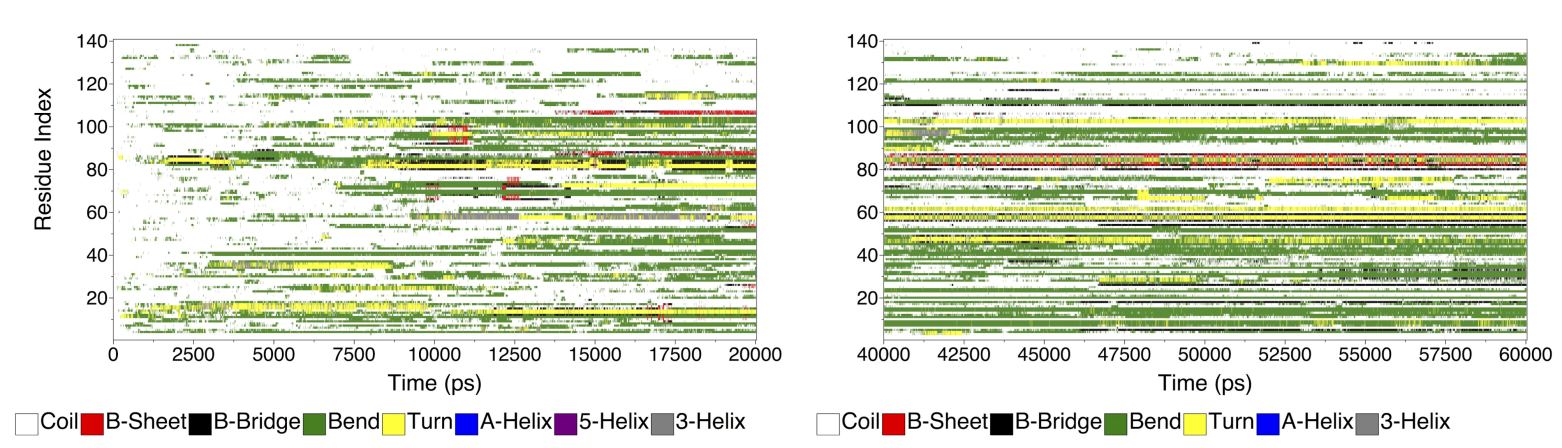
**

**Fig. S2.** Secondary structure evolution of the monomer in the course of a 60 ns simulation. The timelines of the secondary structures in the course of the first 20 ns of the MD simulation (left) and the last 20 ns of the same simulation (right) are shown.

**
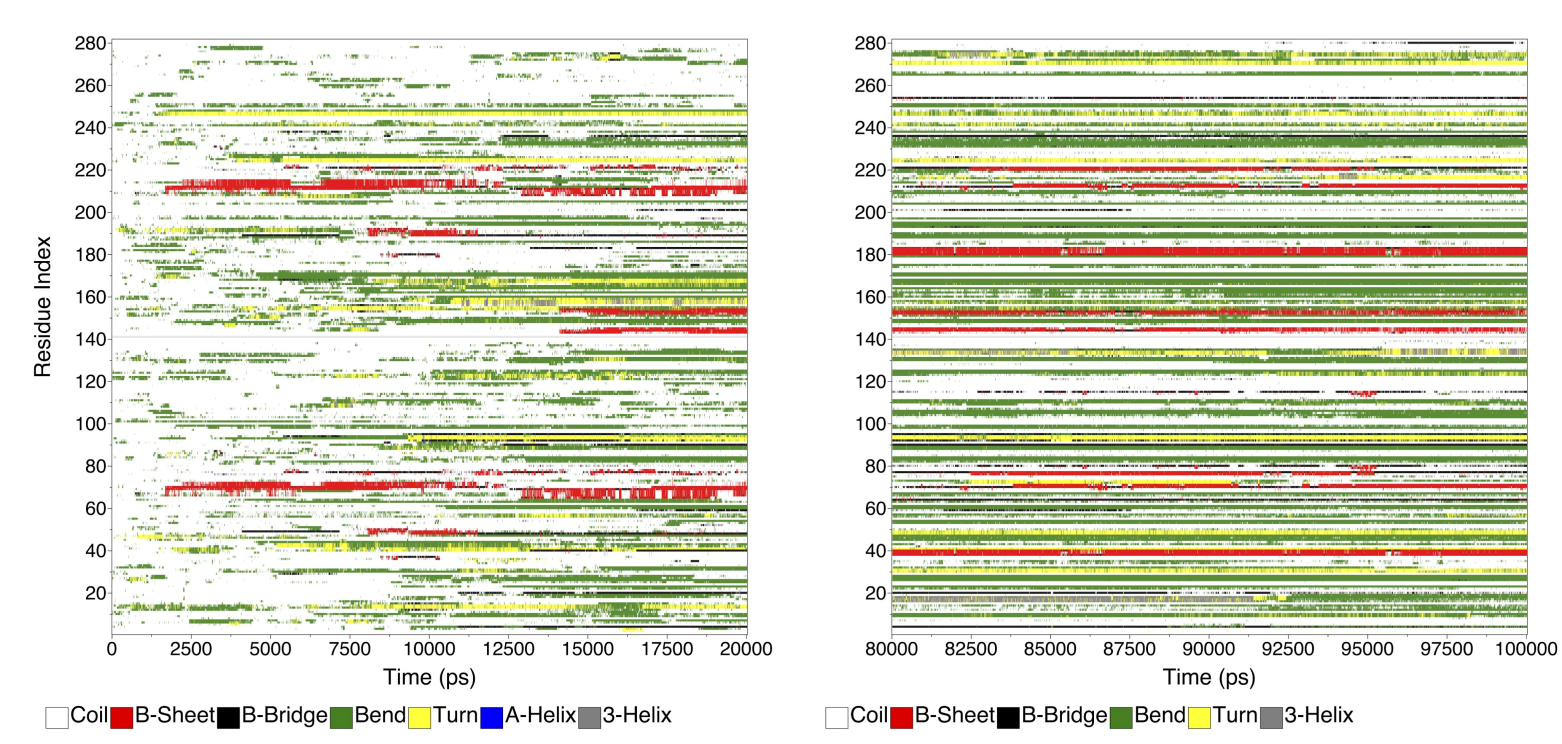
**

**Fig. S3.** Secondary structure evolution of the HH1 dimer in the course of a 100 ns simulation: the timeline of the secondary structure evolution in the course of the first 20 ns showing the formation of secondary structure (left), and the last 20 ns illustrating the stability of the formed secondary structure elements (right).

**
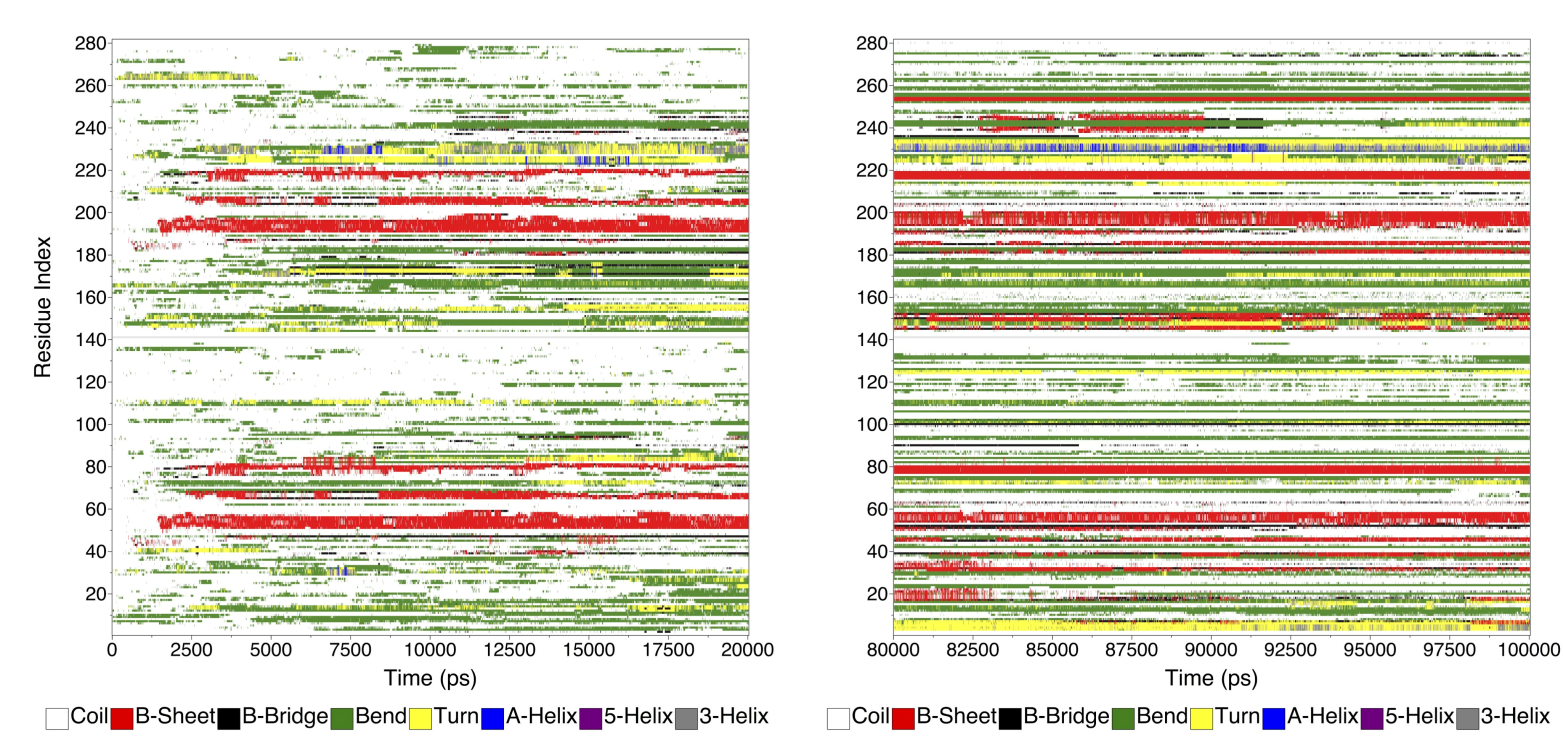
**

**Fig. S4.** Secondary structure evolution of the HH2 dimer in the course of a 100 ns simulation: the timeline of the secondary structure evolution in the course of the first 20 ns showing the formation of secondary structure (left), and the last 20 ns illustrating the stability of the formed secondary structure elements (right).

**
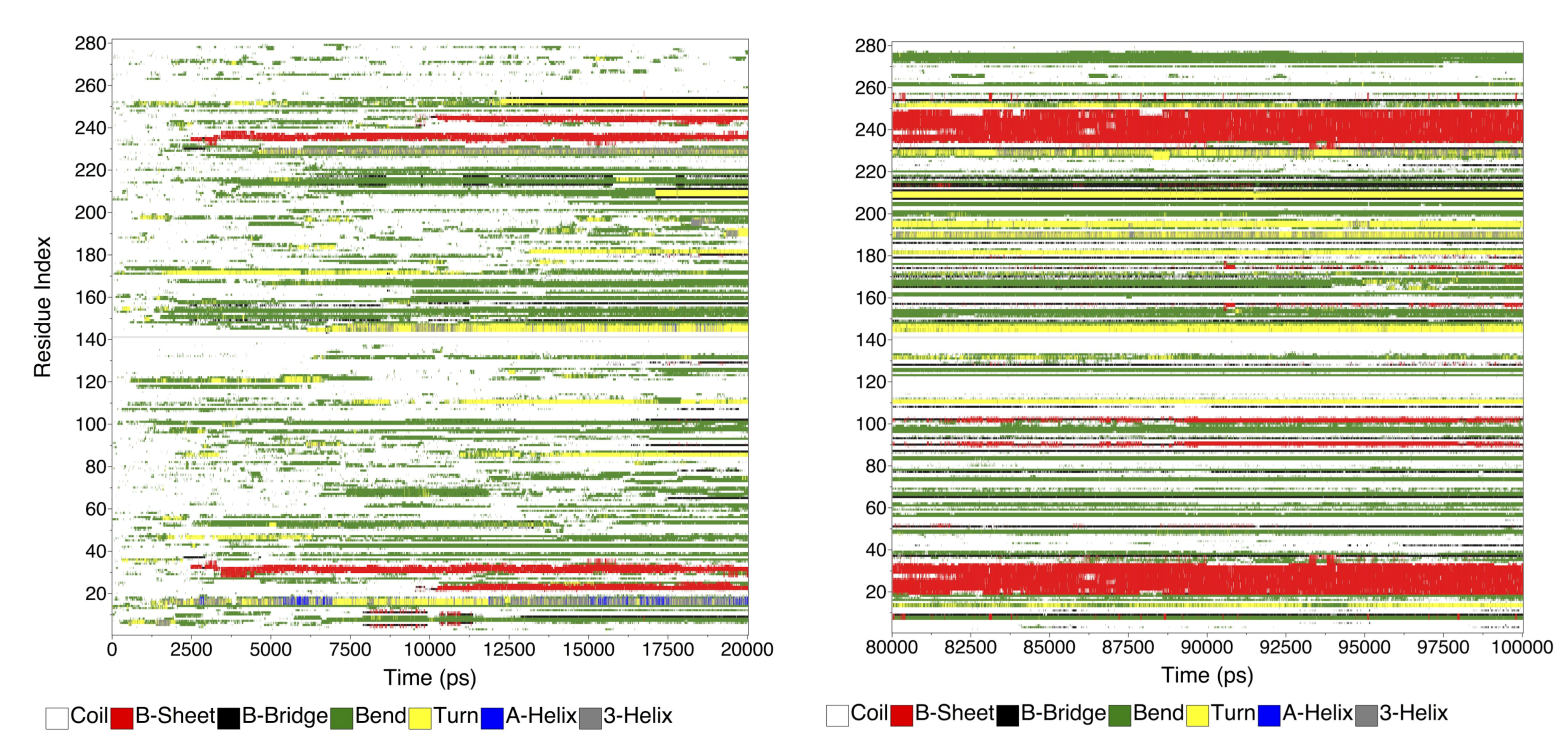
**

**Fig. S5.** Secondary structure evolution of the HT1 dimer for the first 20 ns (left), and the last 20 ns (right) of a 100 ns simulation.

**
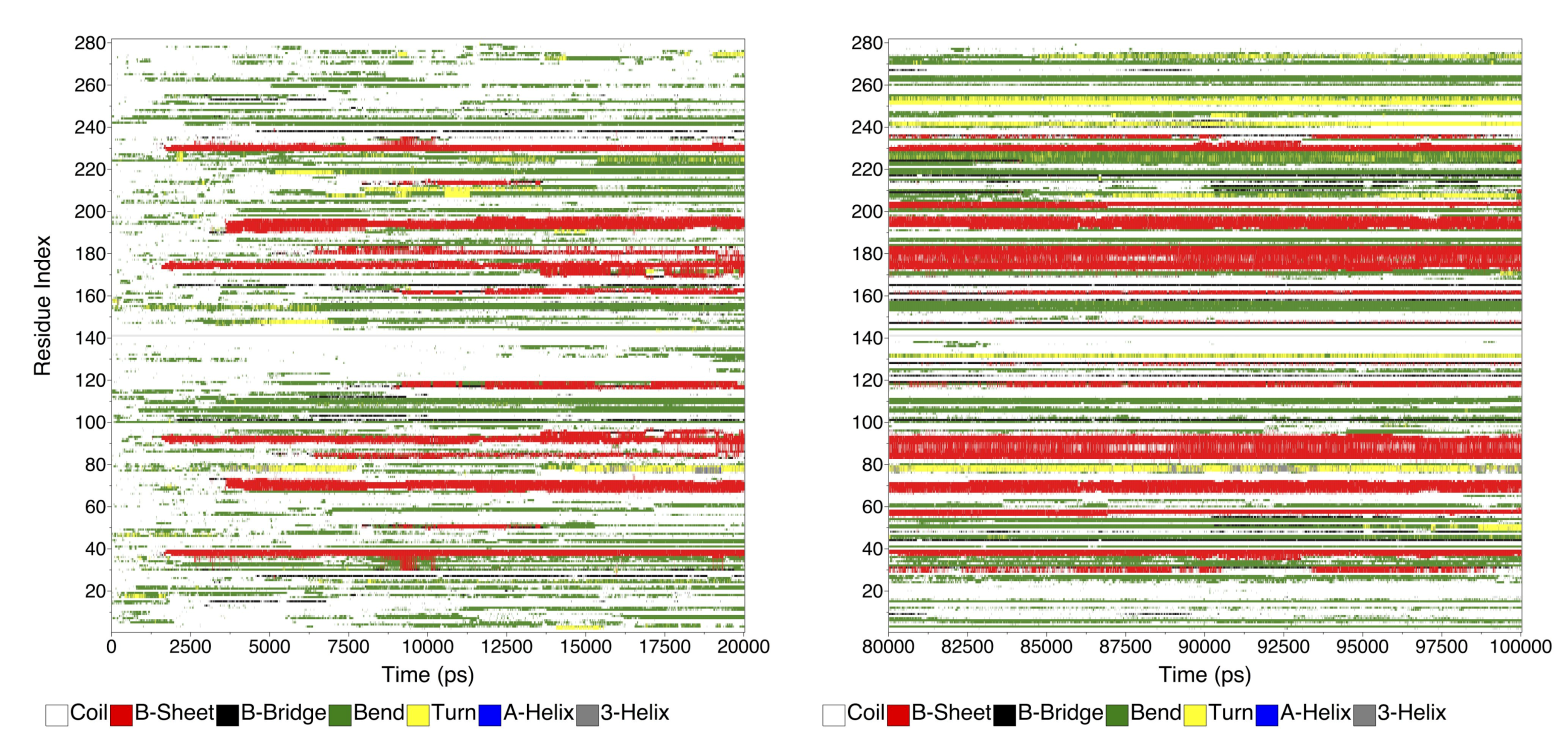
**

**Fig. S6.** Secondary structure evolution of the HT2 dimer for the first 20 ns (left), and the last 20 ns (right) of a 100 ns simulation.


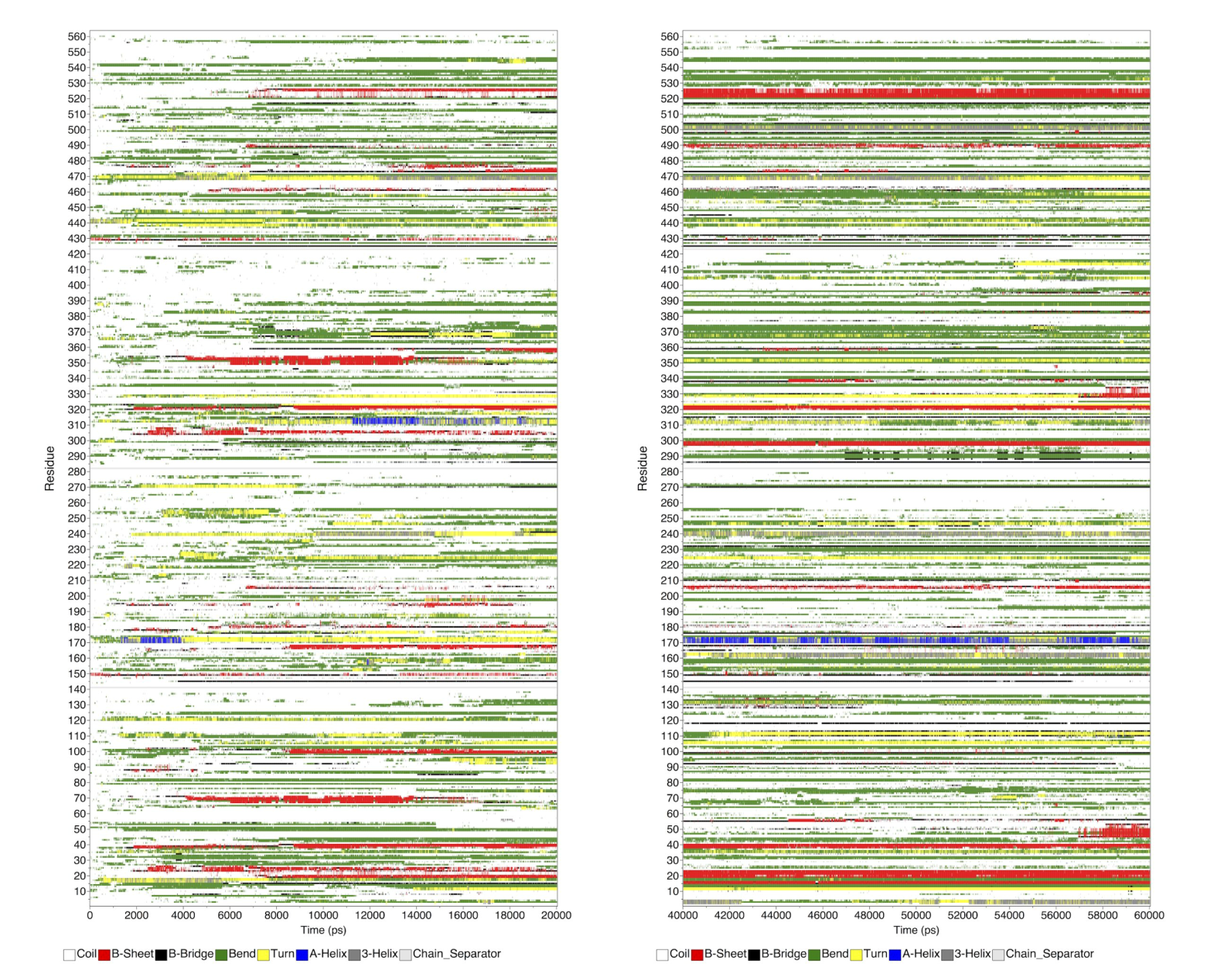


**Fig. S7.** Timeline of the secondary structure evolution of the tetramer in the course of the first 20 ns showing the formation of secondary structure (left), and the last 20 ns illustrating the stability of the formed secondary structure elements (right). The total simulation time is 60 ns.
